# Supplementary figures and images for: Cryo-EM structure revealed a novel F-actin binding motif in a Legionella pneumophila lysine fatty acyltransferase
Source: eLife. 2026 Jan 28;14:RP106975. doi: 10.7554/eLife.106975 (PMC12851578; doi:10.7554/eLife.106975)

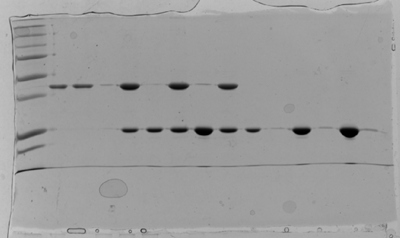

Supplement: Figure 1—source data 1. [file elife-106975-fig1-data1.zip › figure 1F.TIF]

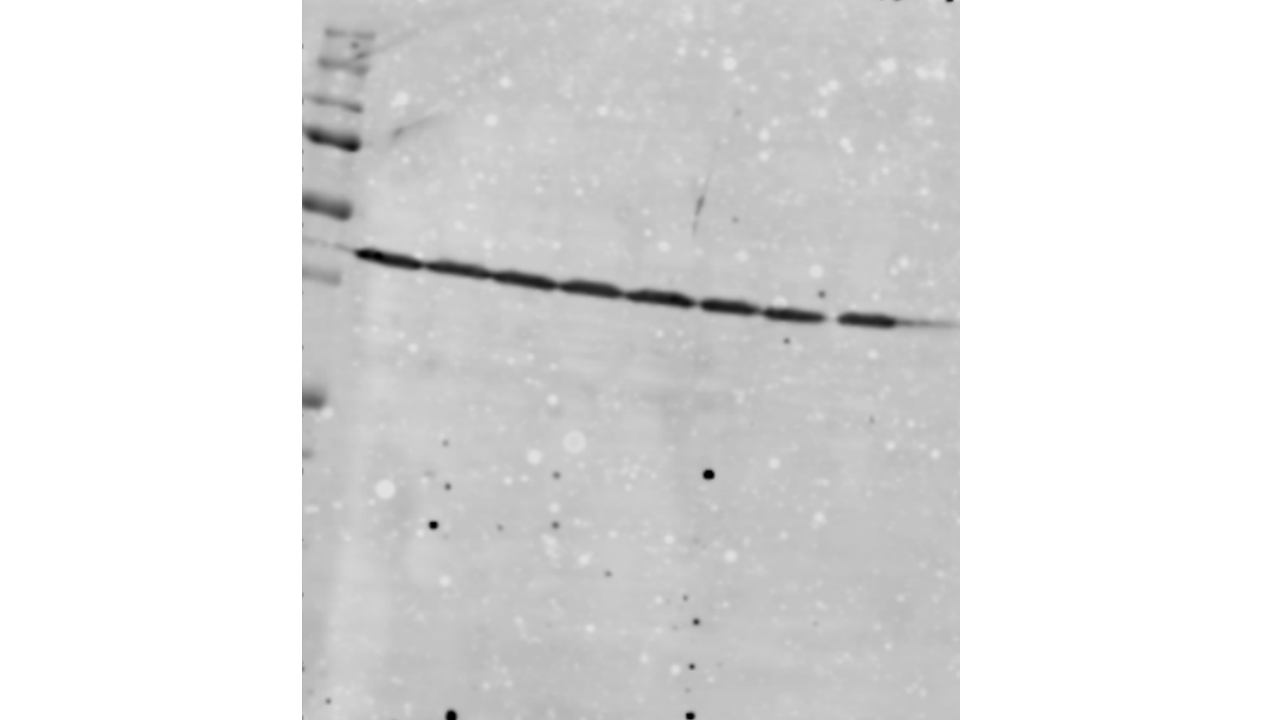

Supplement: Figure 1—source data 1. [file elife-106975-fig1-data1.zip › 1D_Input_Actin.tif]

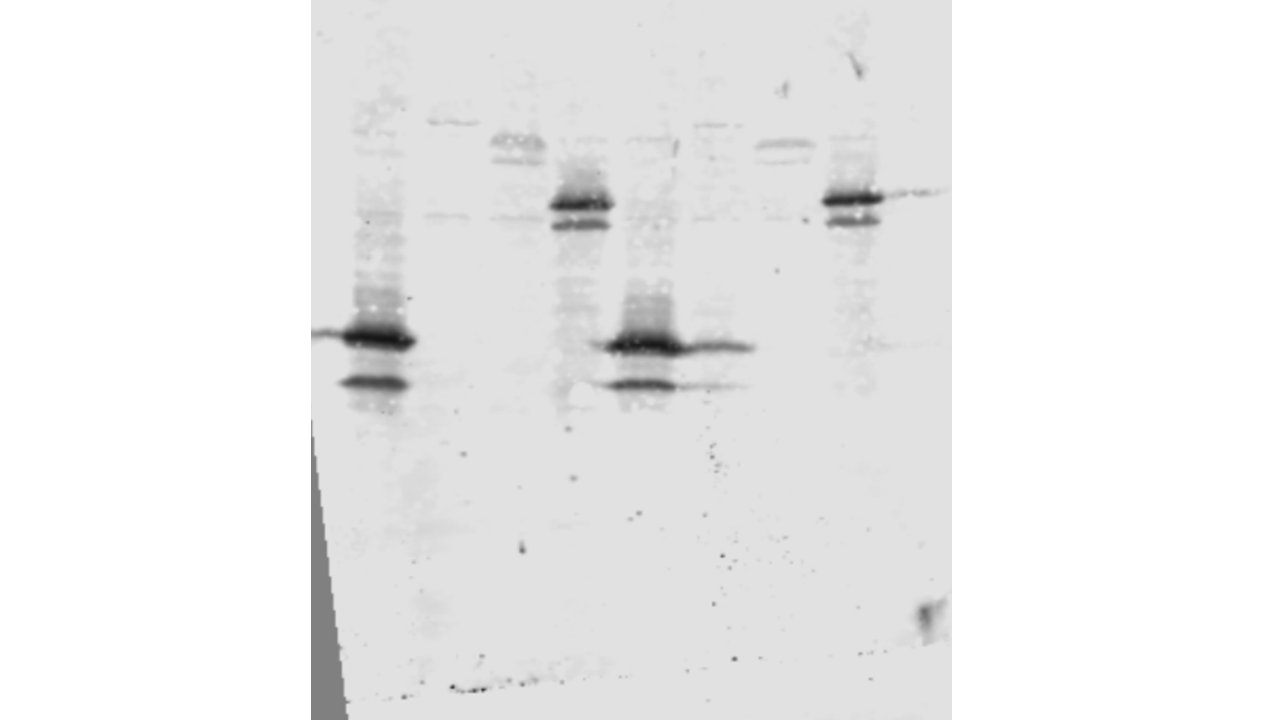

Supplement: Figure 1—source data 1. [file elife-106975-fig1-data1.zip › 1D_Input_GFP.tif]

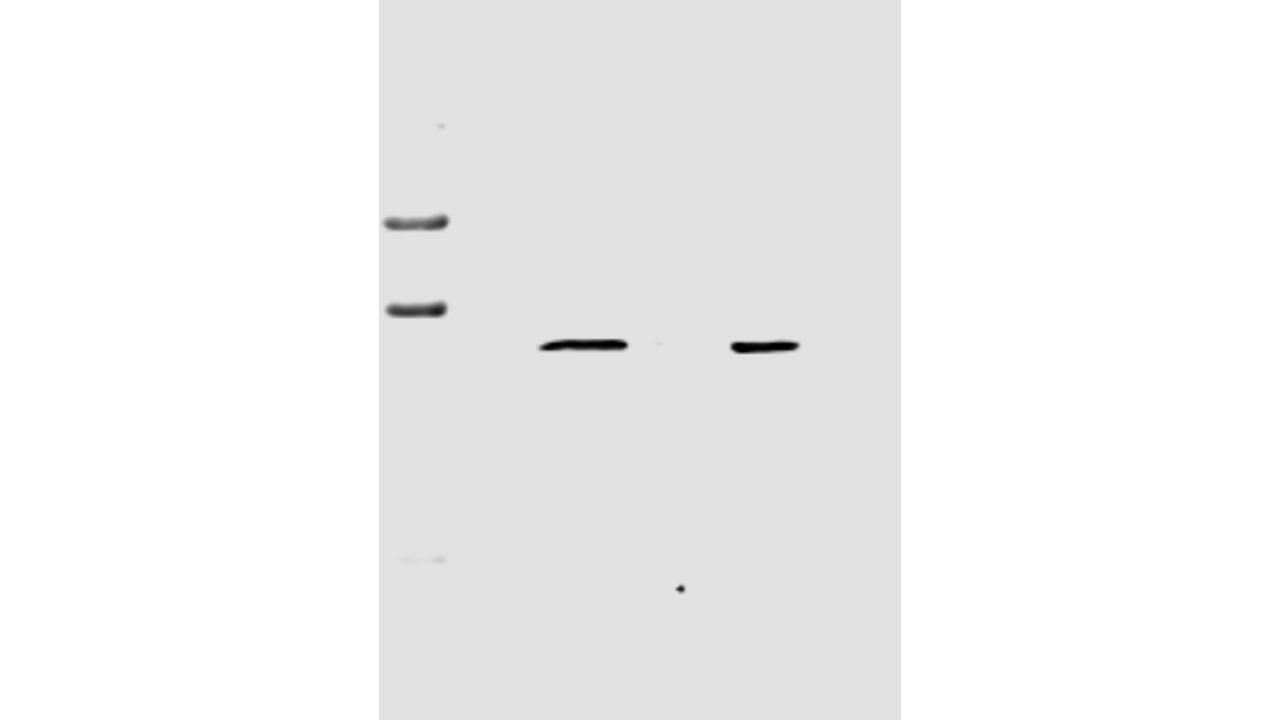

Supplement: Figure 1—source data 1. [file elife-106975-fig1-data1.zip › 1D_IP_Actin.tif]

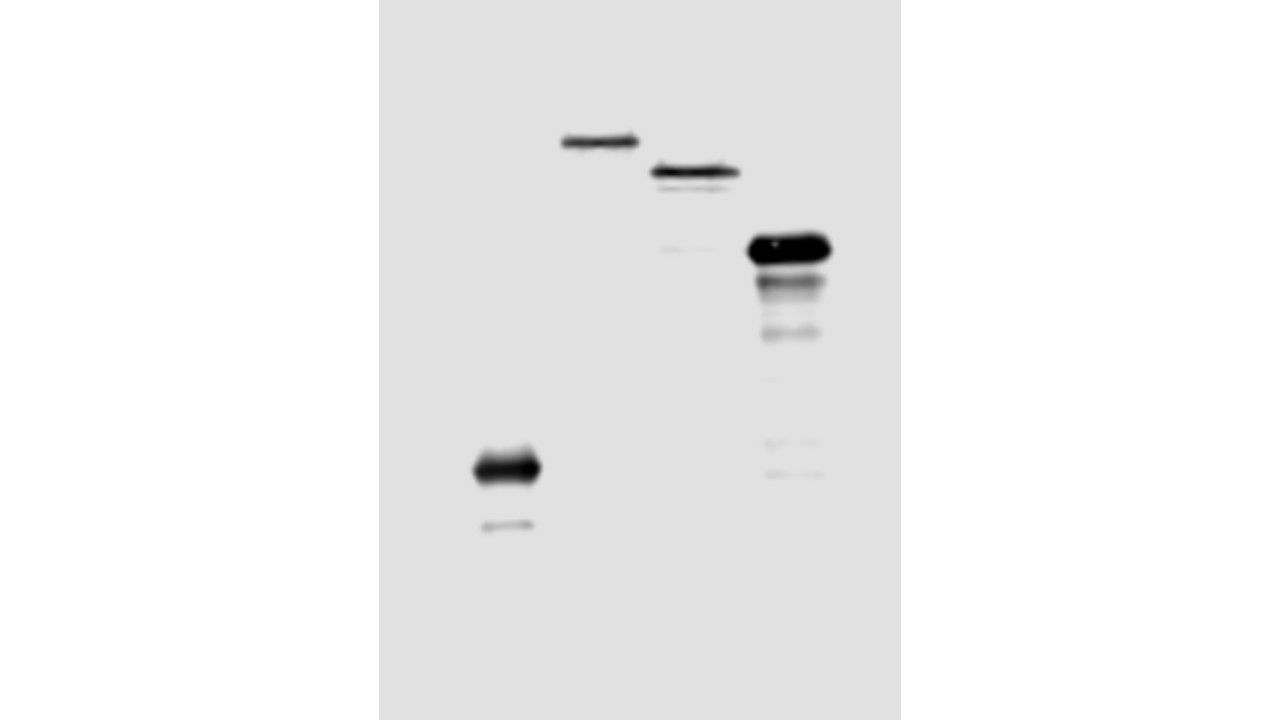

Supplement: Figure 1—source data 1. [file elife-106975-fig1-data1.zip › 1D_IP_GFP.tif]

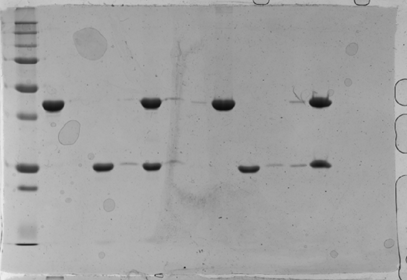

Supplement: Figure 1—source data 1. [file elife-106975-fig1-data1.zip › figure 1E.tif]

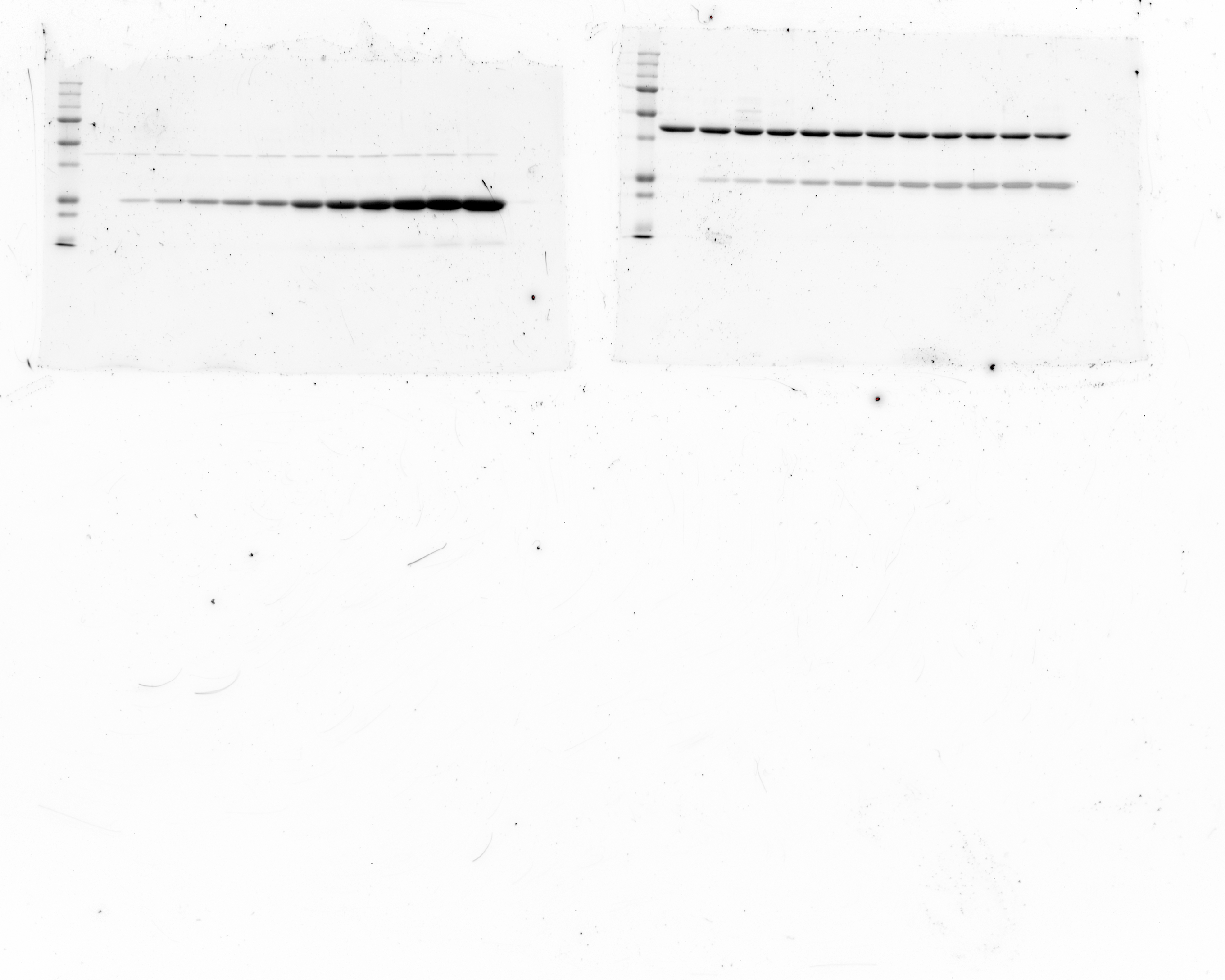

Supplement: Figure 3—source data 1. [file elife-106975-fig3-data1.zip › Figure3D_R236A_Run3.jpg]

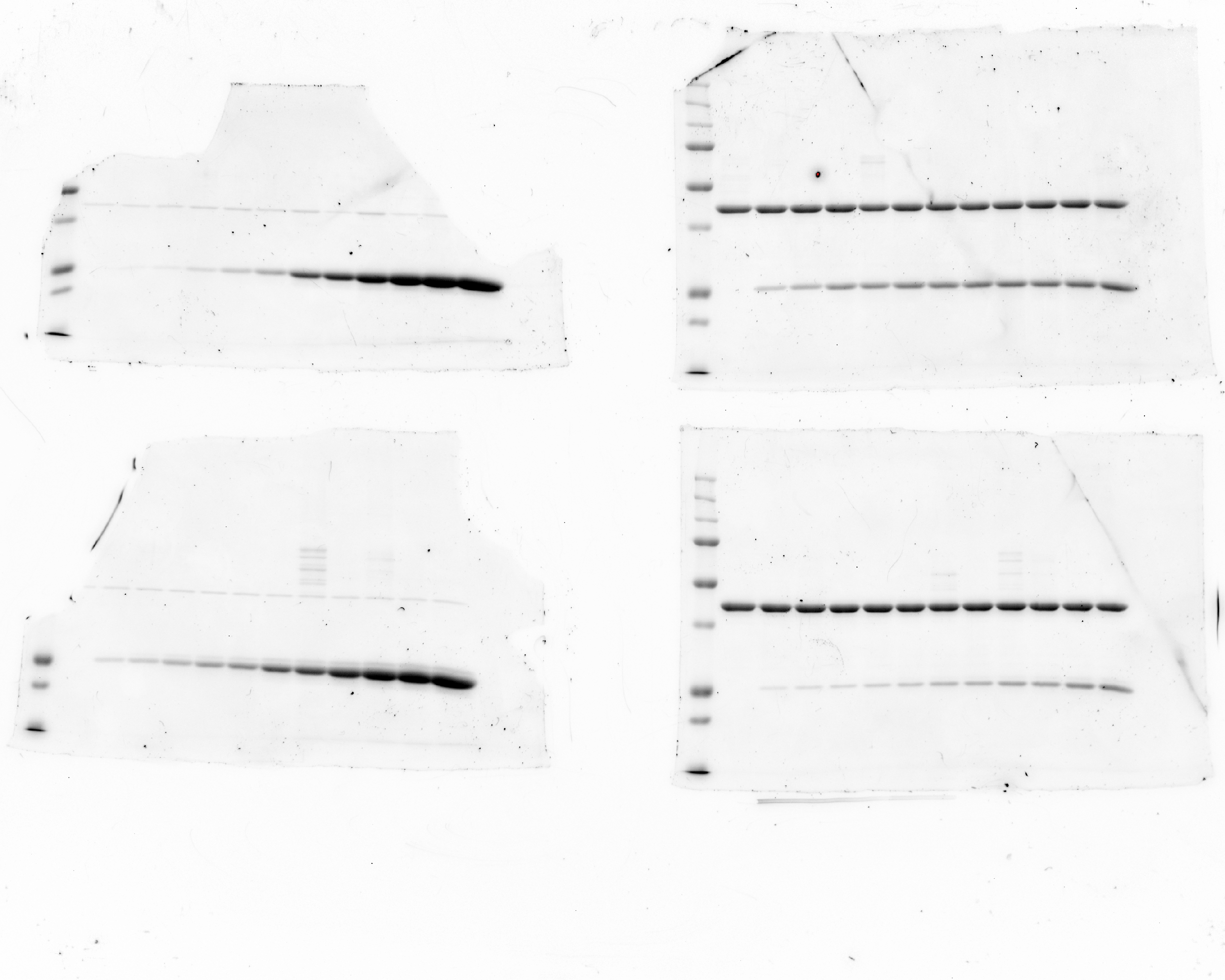

Supplement: Figure 3—source data 1. [file elife-106975-fig3-data1.zip › Figure3D_WT&Y240A_Run2.jpg]

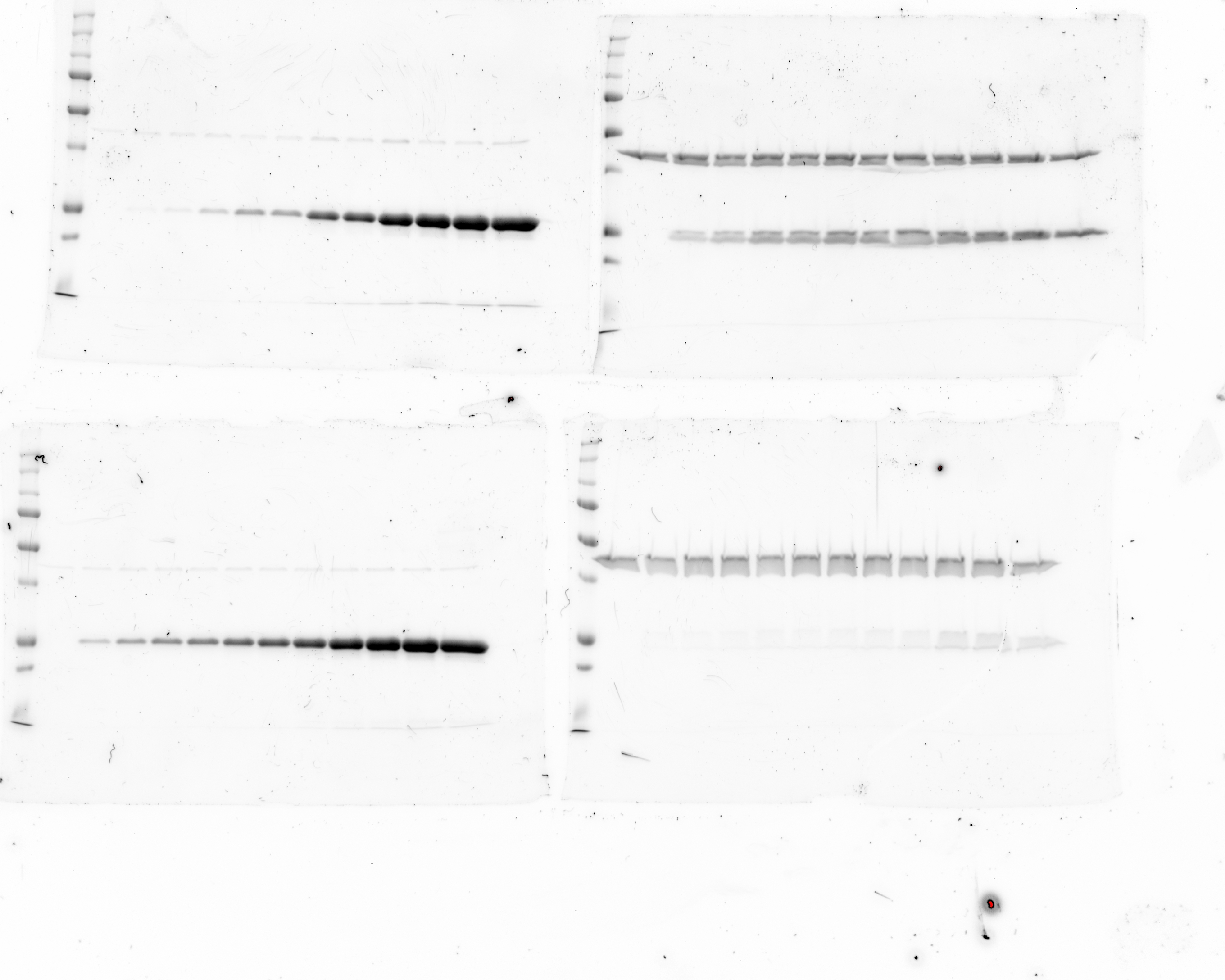

Supplement: Figure 3—source data 1. [file elife-106975-fig3-data1.zip › Figure3D_WT&Y240A_Run3.jpg]

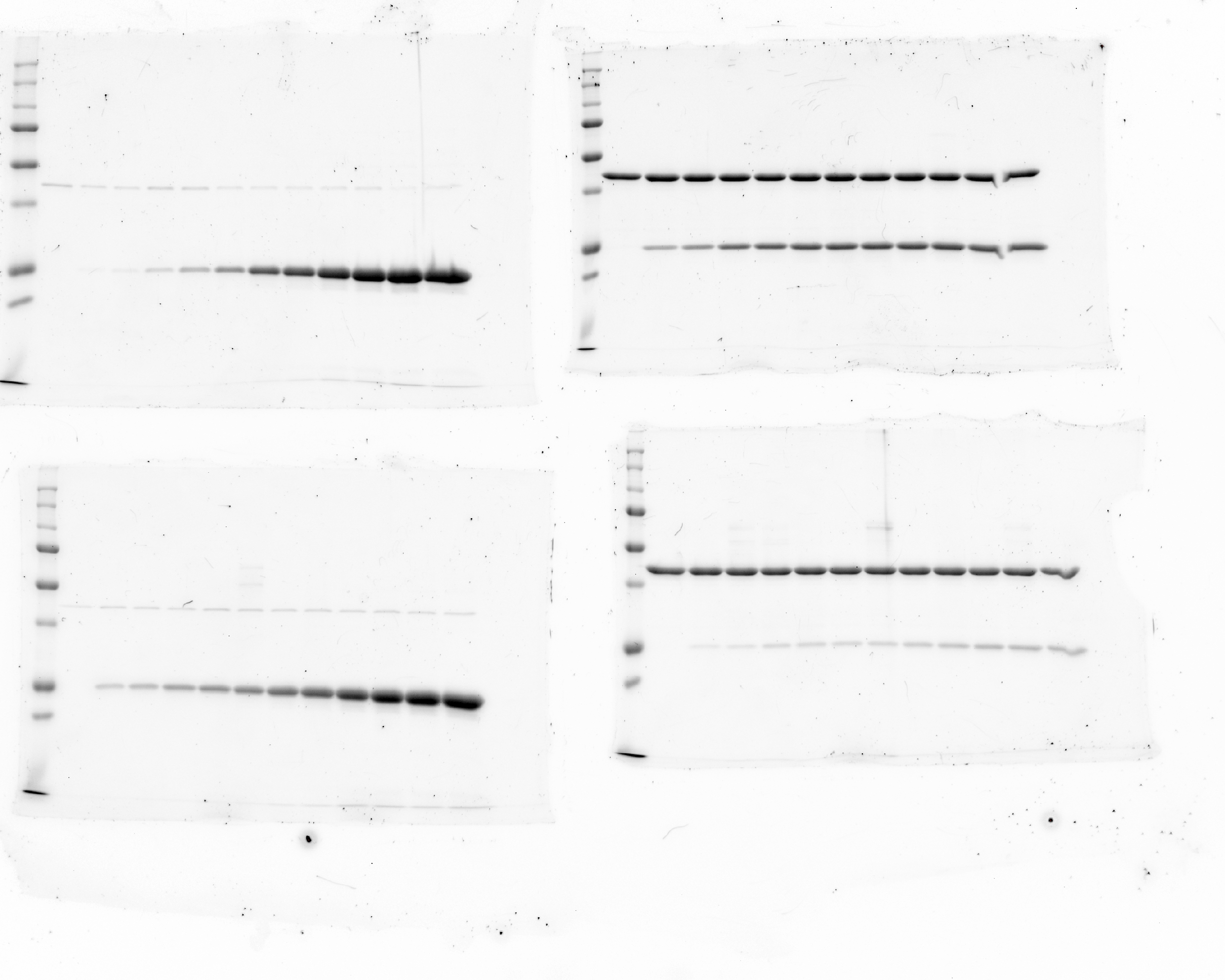

Supplement: Figure 3—source data 1. [file elife-106975-fig3-data1.zip › Figure 3D_WT&Y240A_Run1.jpg]

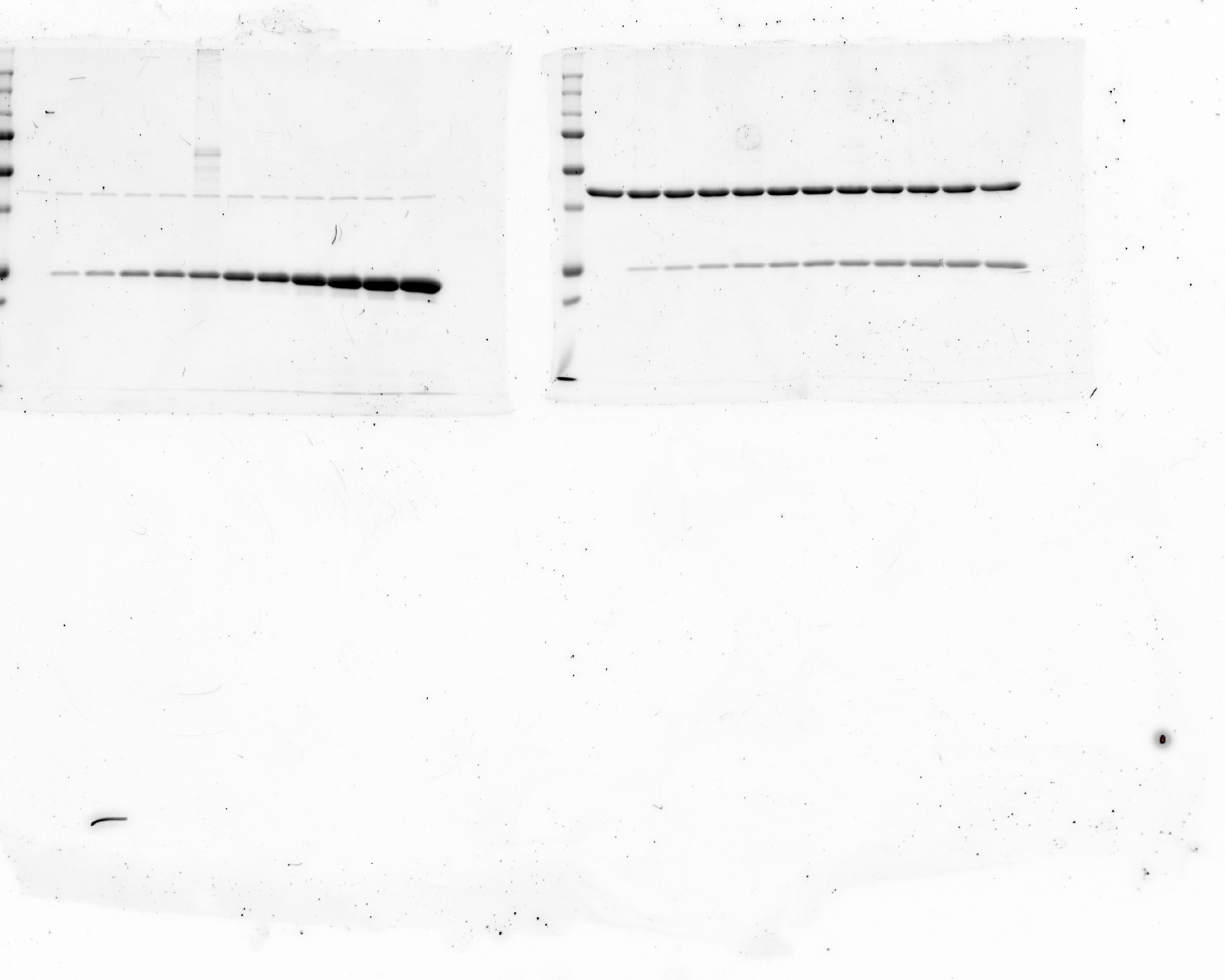

Supplement: Figure 3—source data 1. [file elife-106975-fig3-data1.zip › Figure3D_Q254A_Run1.jpg]

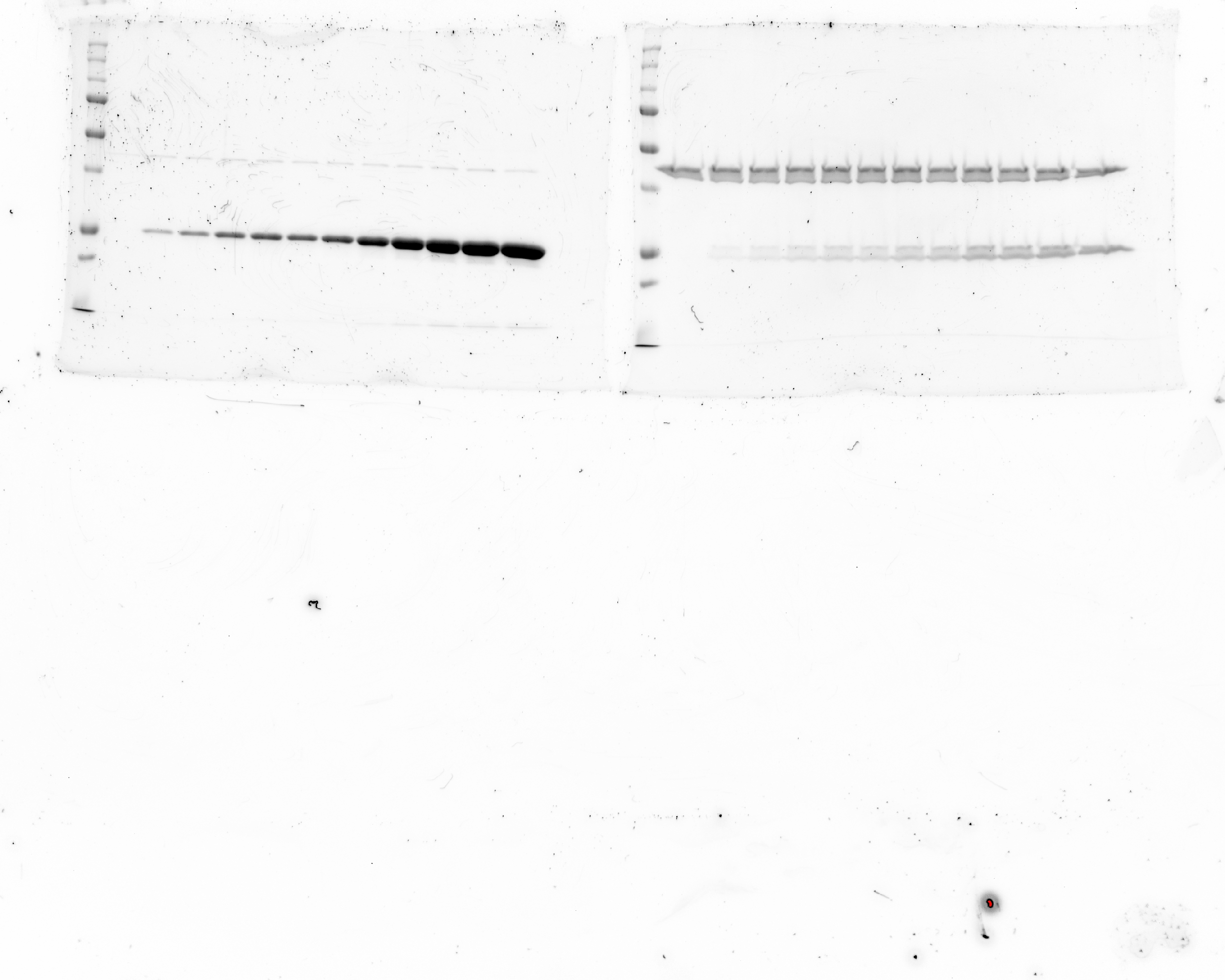

Supplement: Figure 3—source data 1. [file elife-106975-fig3-data1.zip › Figure3D_Q254A_Run2.jpg]

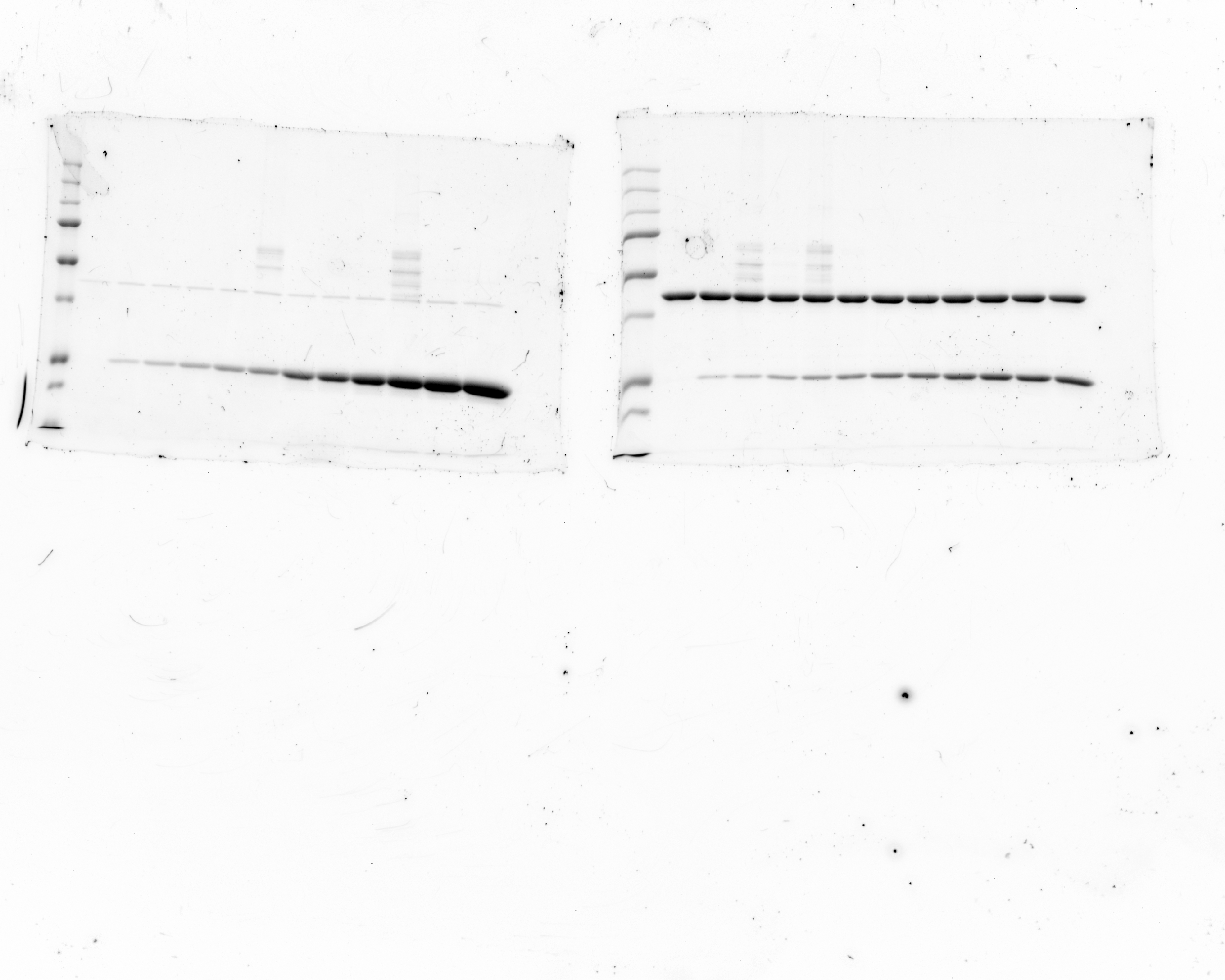

Supplement: Figure 3—source data 1. [file elife-106975-fig3-data1.zip › Figure3D_Q254A_Run3.jpg]

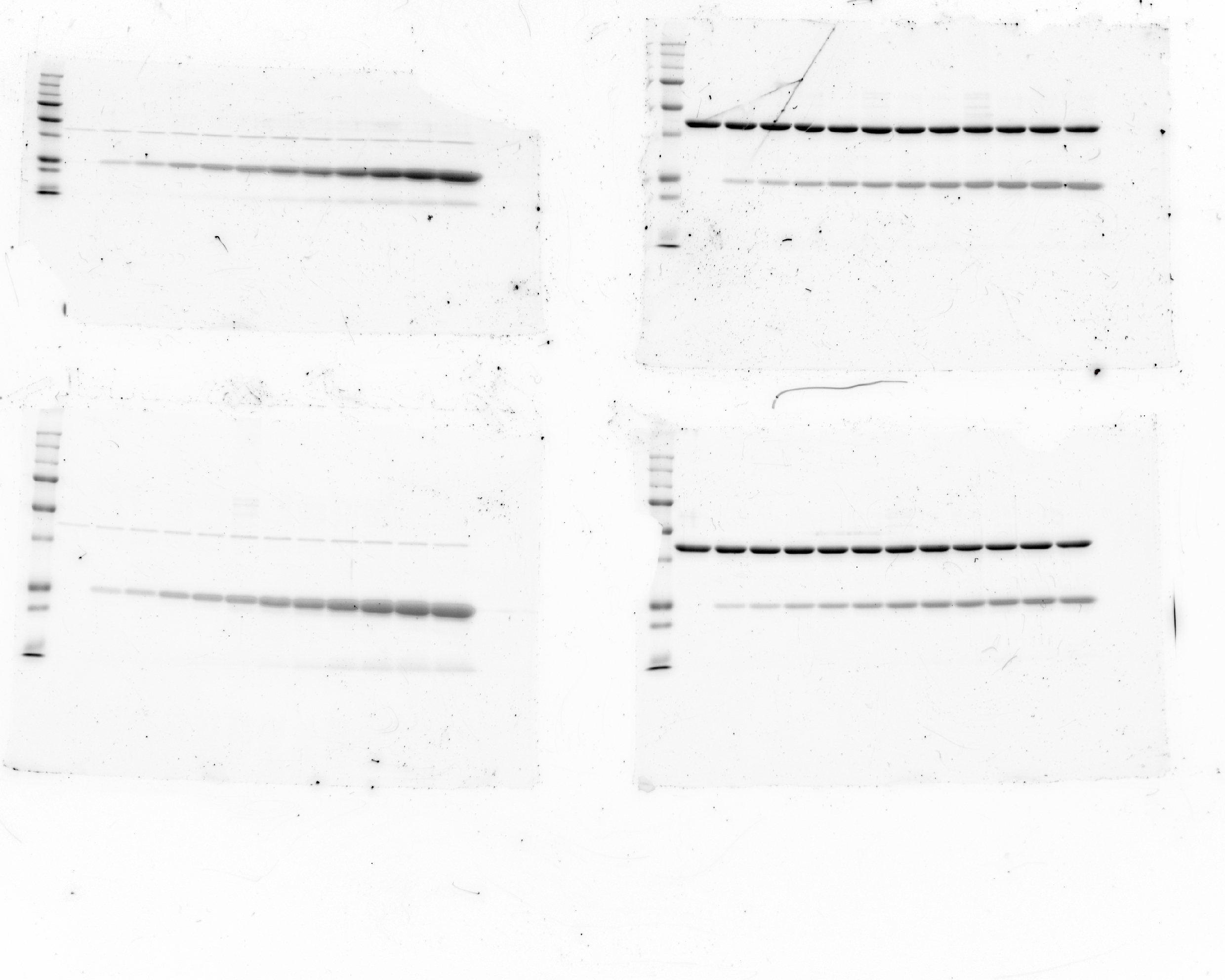

Supplement: Figure 3—source data 1. [file elife-106975-fig3-data1.zip › Figure3D_R236A_Run1&2.jpg]

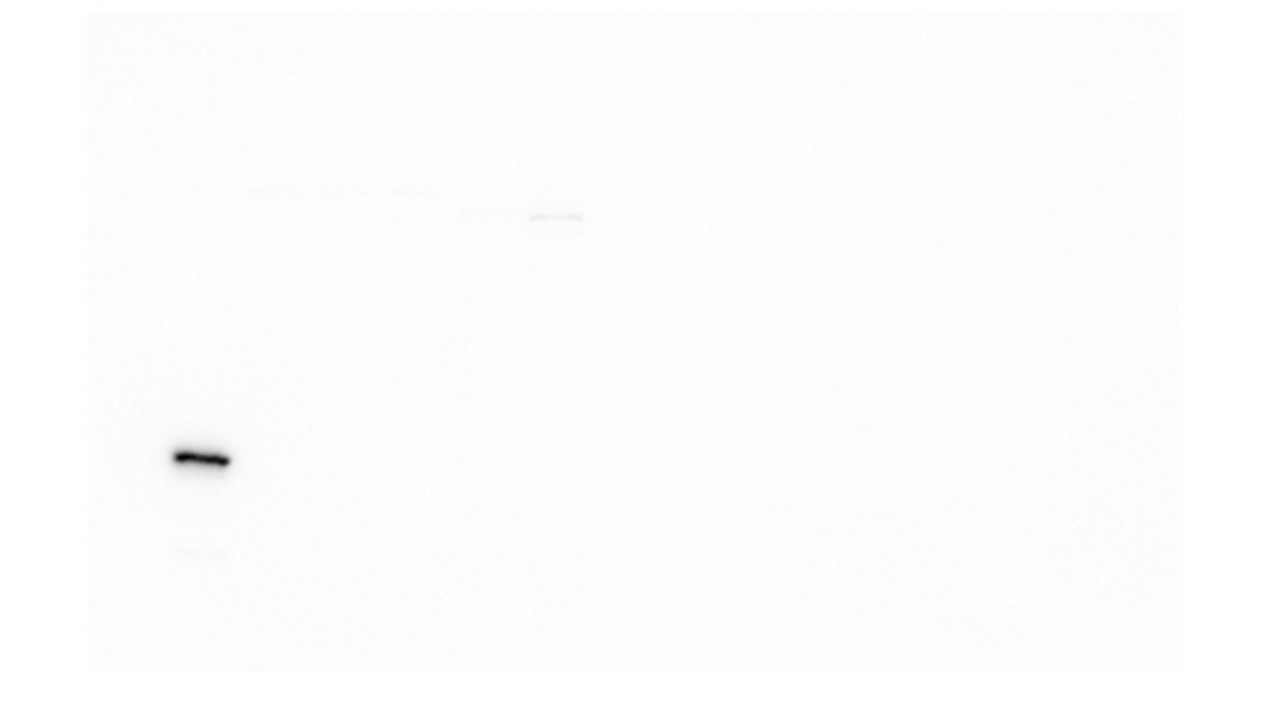

Supplement: Figure 6—source data 1. [file elife-106975-fig6-data1.zip › Figure6B_GFPChannel_ShortExposure.tif]

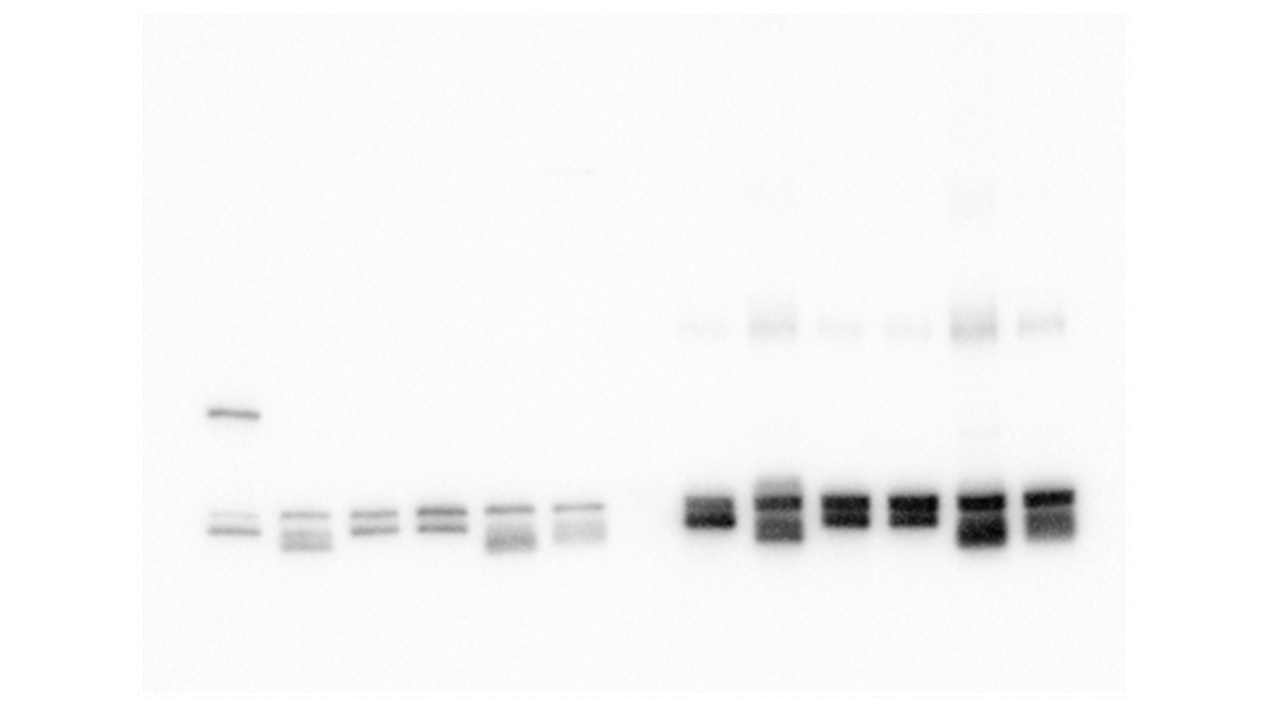

Supplement: Figure 6—source data 1. [file elife-106975-fig6-data1.zip › Figure6B_FlagChannel.tif]

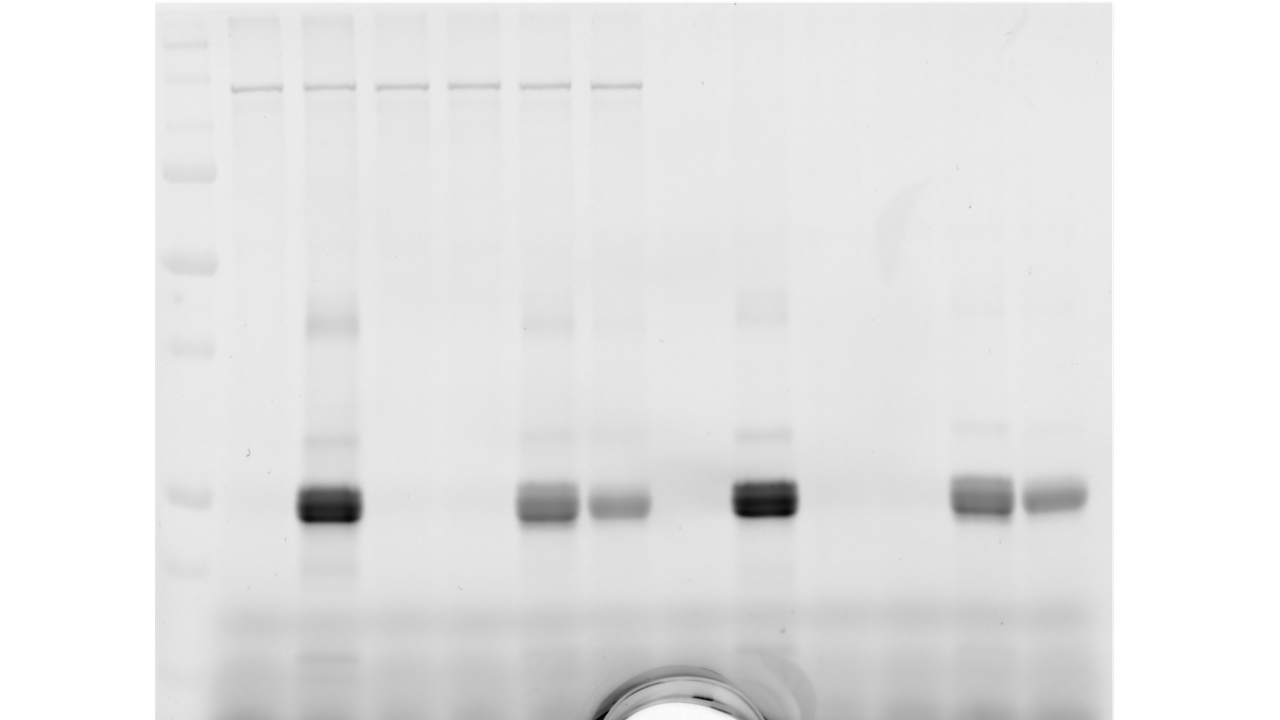

Supplement: Figure 6—source data 1. [file elife-106975-fig6-data1.zip › Figure6B_Fluor.tif]

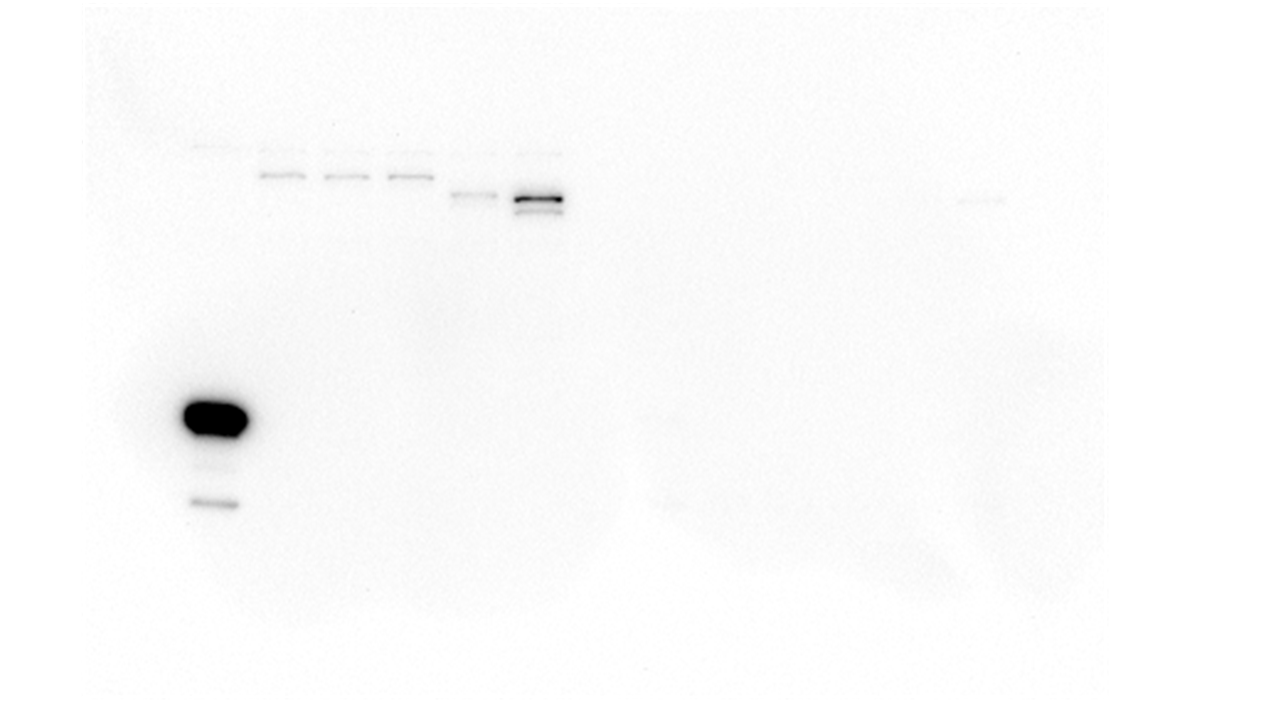

Supplement: Figure 6—source data 1. [file elife-106975-fig6-data1.zip › Figure6B_GFPChannel_LongExposure.tif]

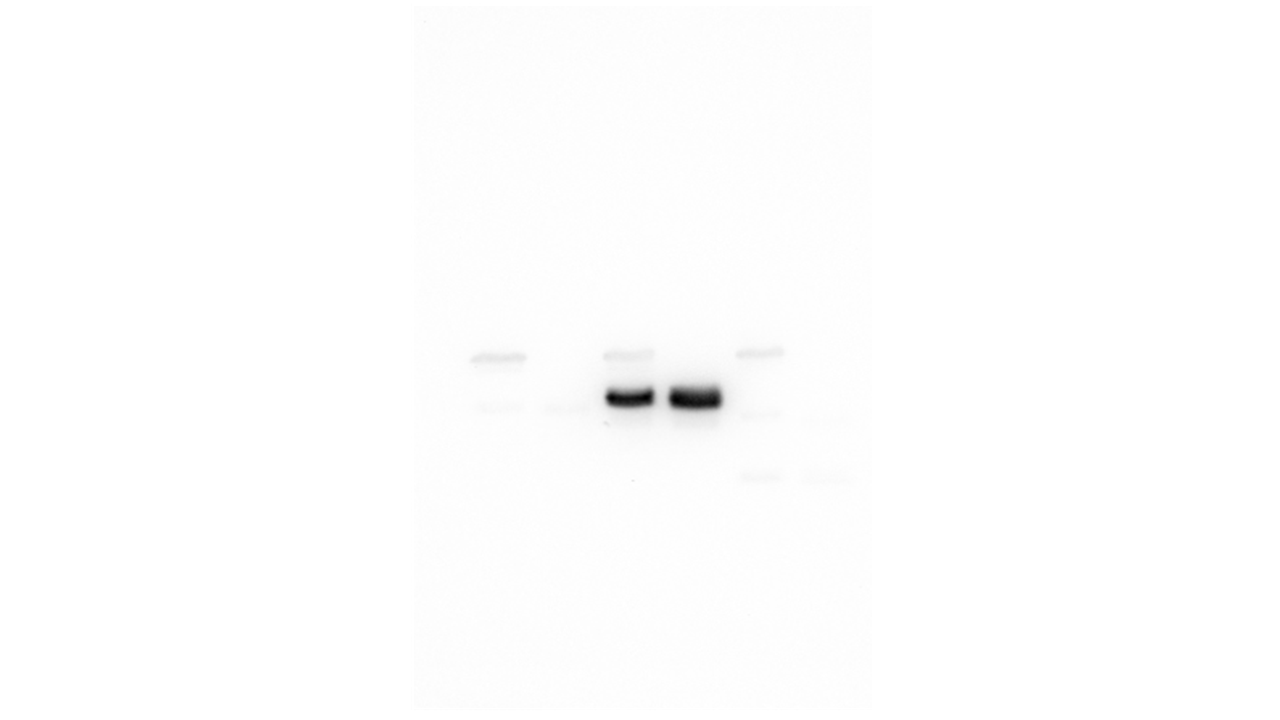

Supplement: Figure 6—figure supplement 1—source data 1. [file elife-106975-fig6-figsupp1-data1.zip › Figure 6-figure supplement 1E_FlagChannel.tif]

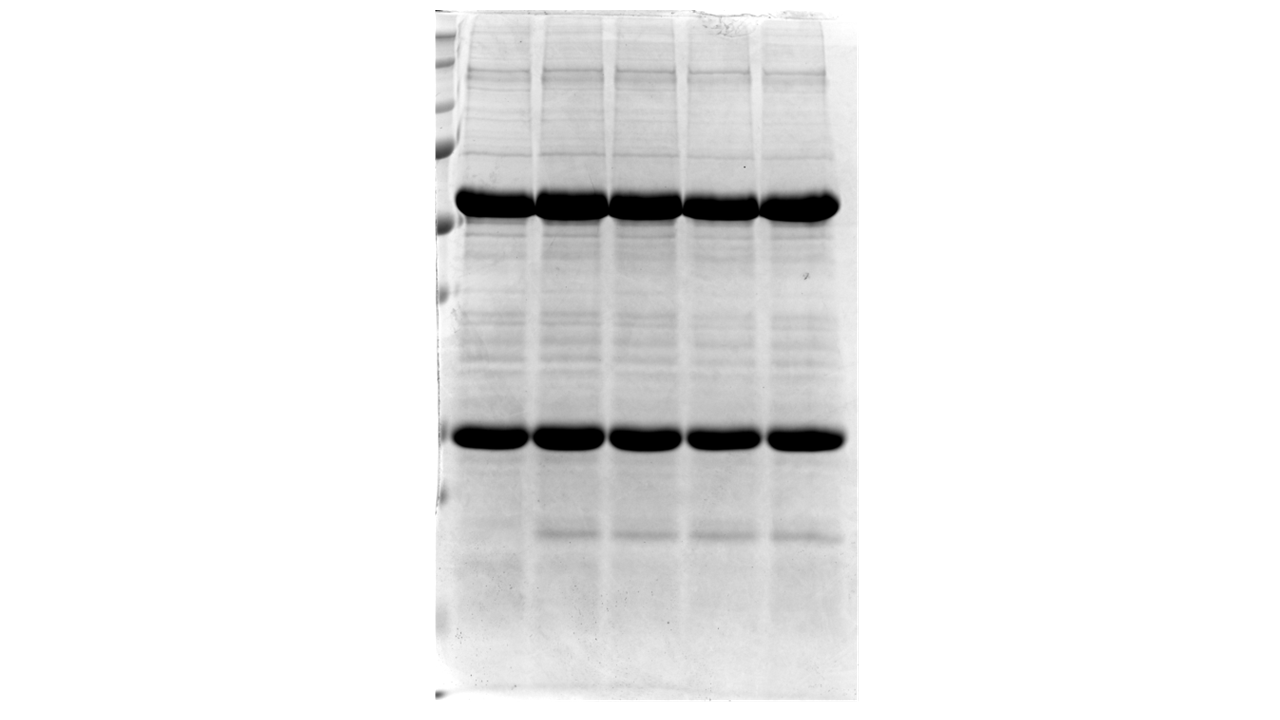

Supplement: Figure 6—figure supplement 1—source data 1. [file elife-106975-fig6-figsupp1-data1.zip › Figure 6-figure supplement 1C_CBB.tif]

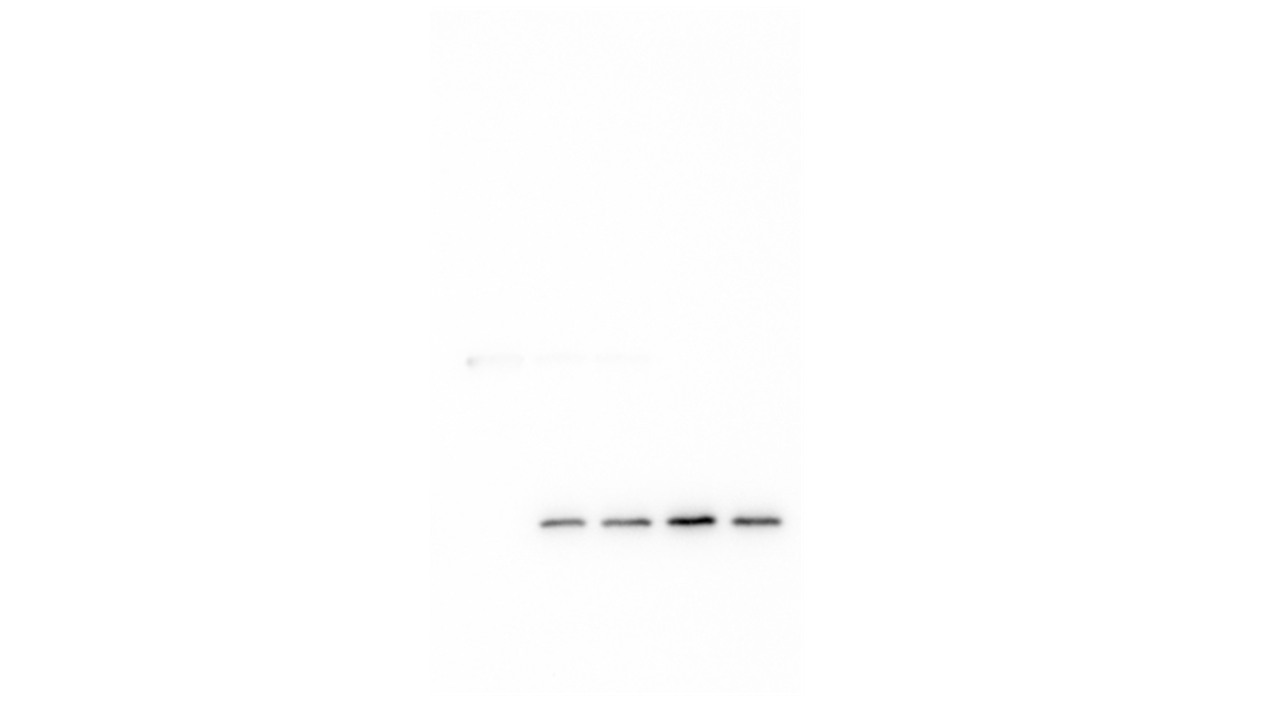

Supplement: Figure 6—figure supplement 1—source data 1. [file elife-106975-fig6-figsupp1-data1.zip › Figure 6-figure supplement 1C_Flag.tif]

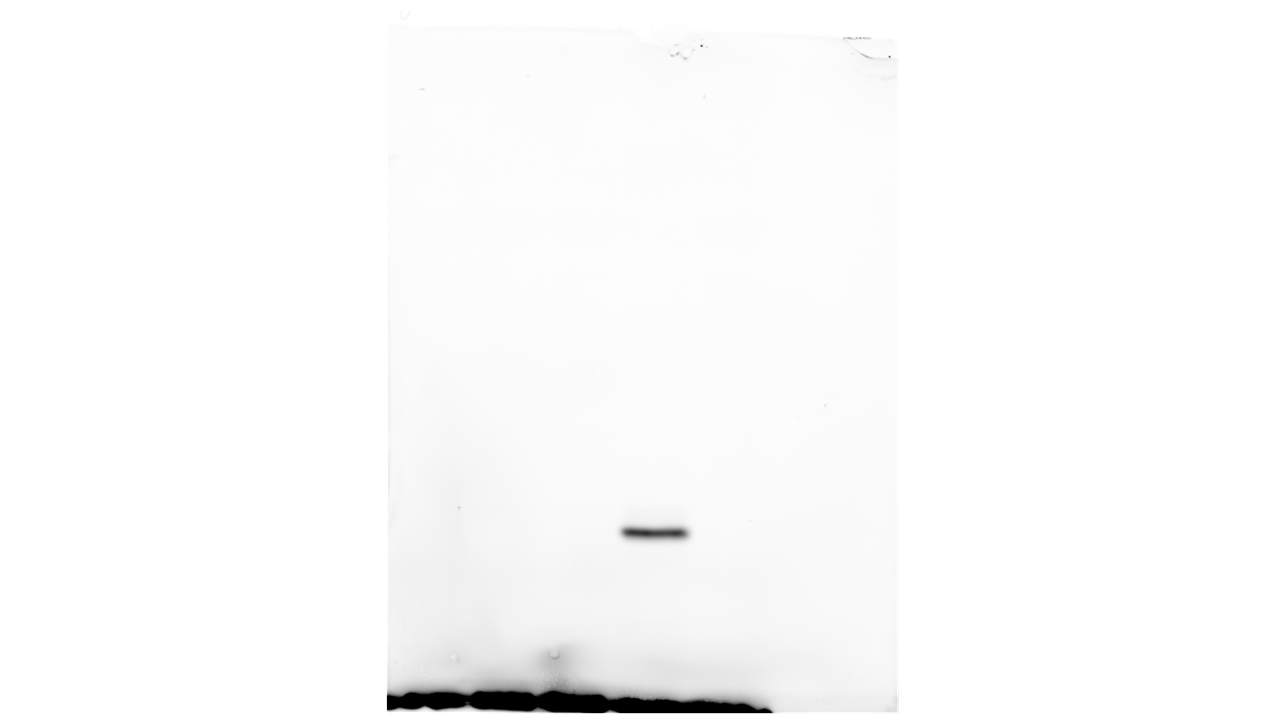

Supplement: Figure 6—figure supplement 1—source data 1. [file elife-106975-fig6-figsupp1-data1.zip › Figure 6-figure supplement 1C_Fluor.tif]

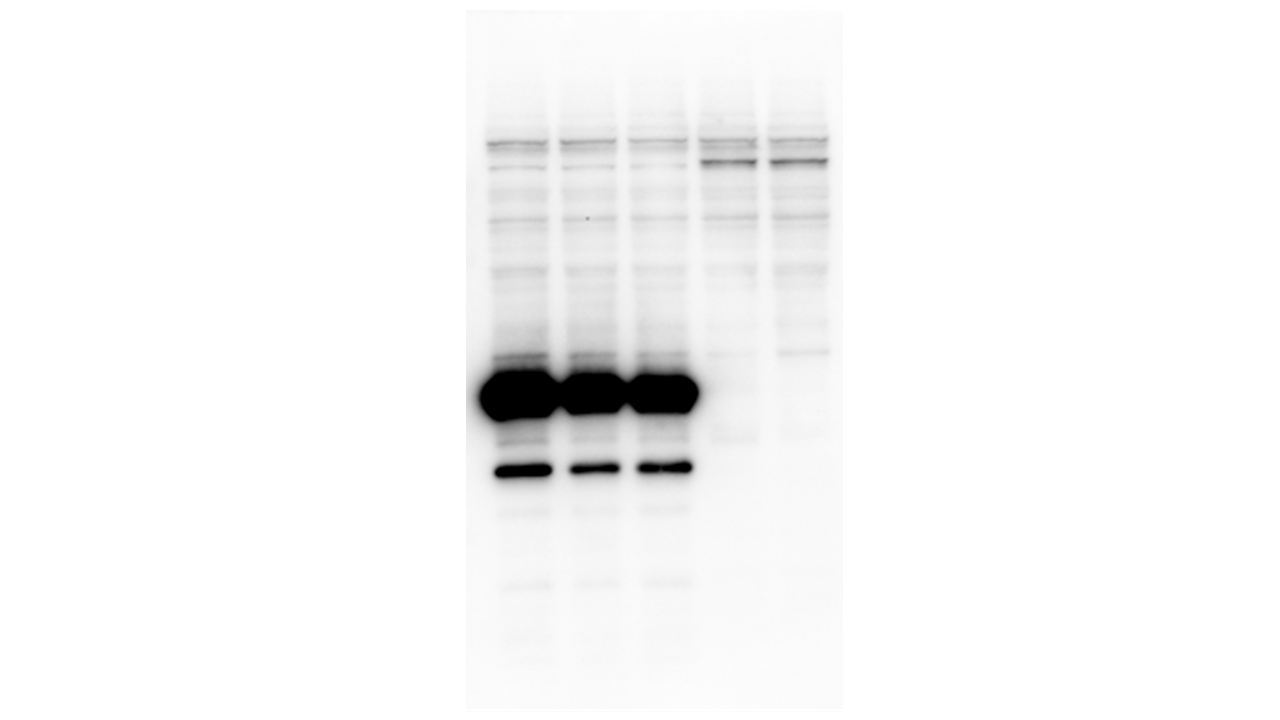

Supplement: Figure 6—figure supplement 1—source data 1. [file elife-106975-fig6-figsupp1-data1.zip › Figure 6-figure supplement 1C_GFP.tif]

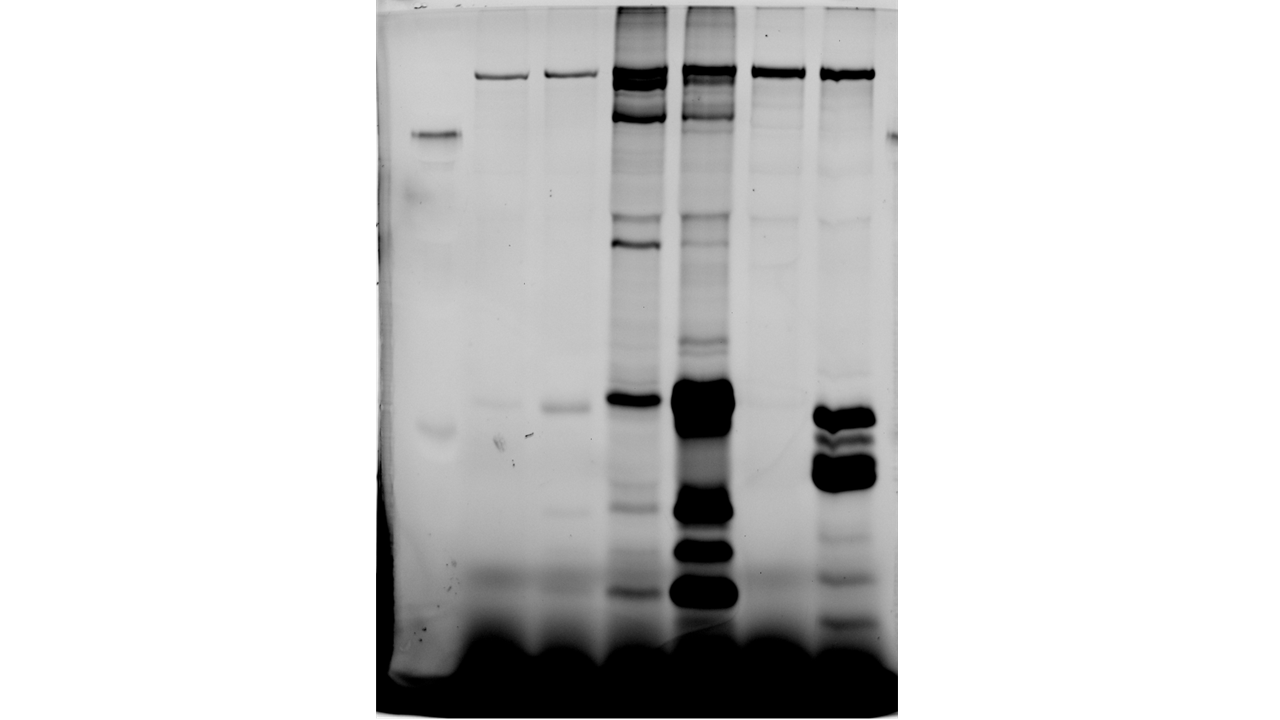

Supplement: Figure 6—figure supplement 1—source data 1. [file elife-106975-fig6-figsupp1-data1.zip › Figure 6-figure supplement 1D, E, F_Fluor_LongExposure.tif]

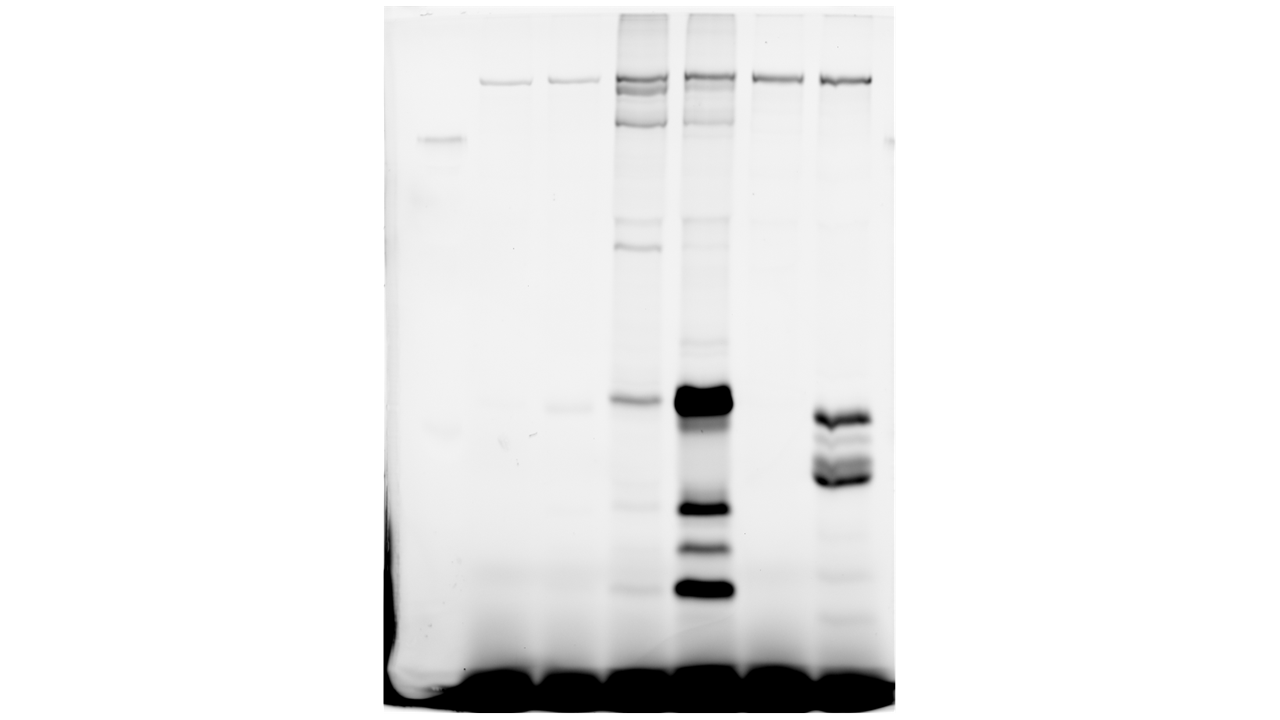

Supplement: Figure 6—figure supplement 1—source data 1. [file elife-106975-fig6-figsupp1-data1.zip › Figure 6-figure supplement 1D, E, F_Fluor_MediumExposure.tif]

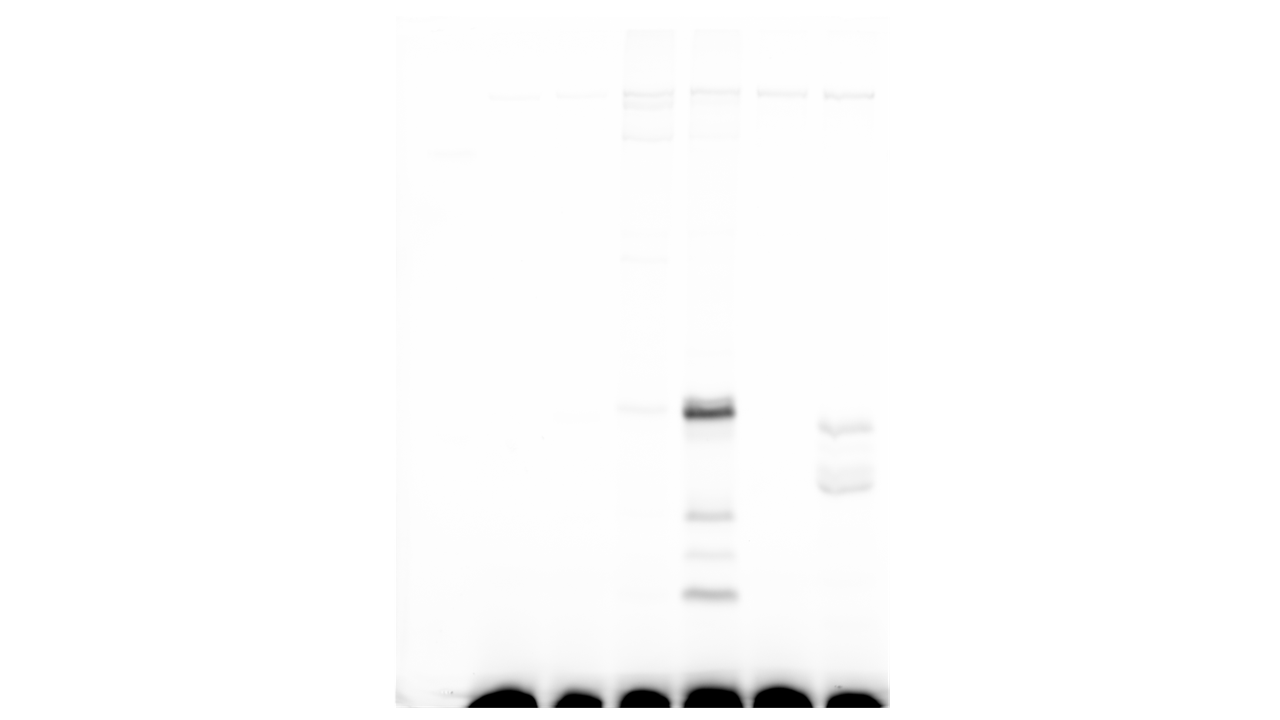

Supplement: Figure 6—figure supplement 1—source data 1. [file elife-106975-fig6-figsupp1-data1.zip › Figure 6-figure supplement 1D, E, F_Fluor_ShortExposure.tif]

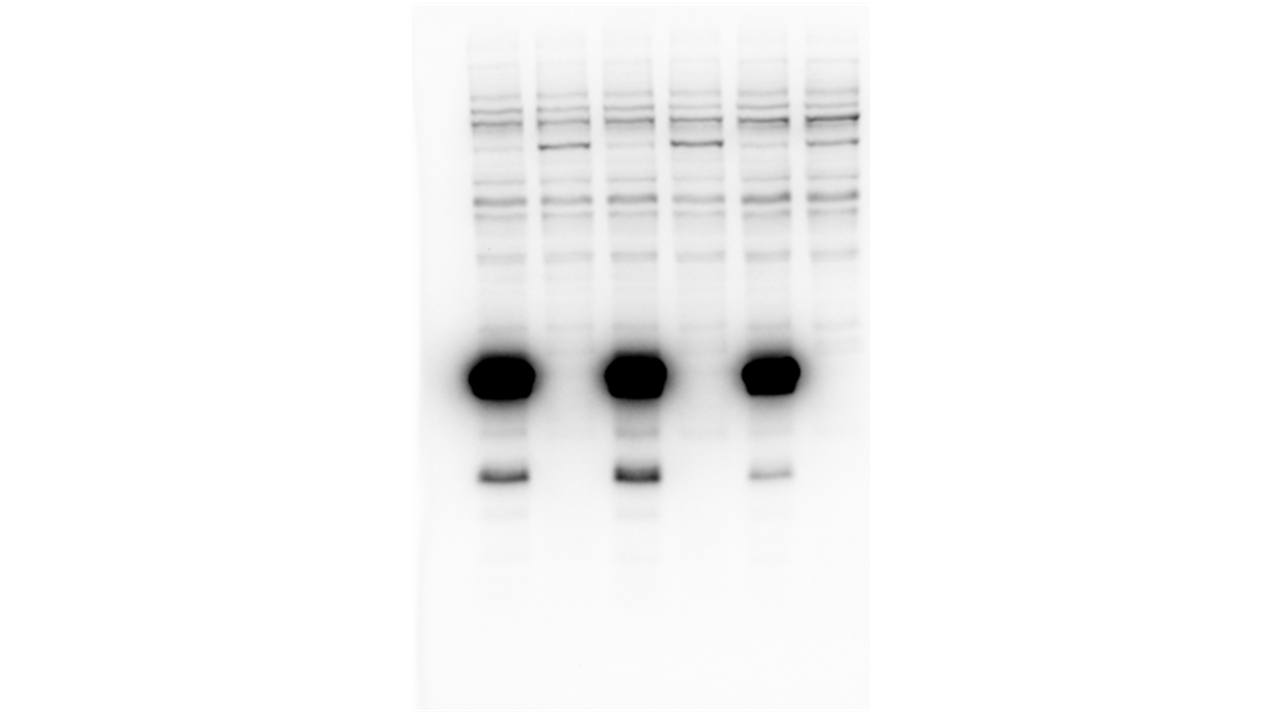

Supplement: Figure 6—figure supplement 1—source data 1. [file elife-106975-fig6-figsupp1-data1.zip › Figure 6-figure supplement 1D, E, F_GFPChannel.tif]

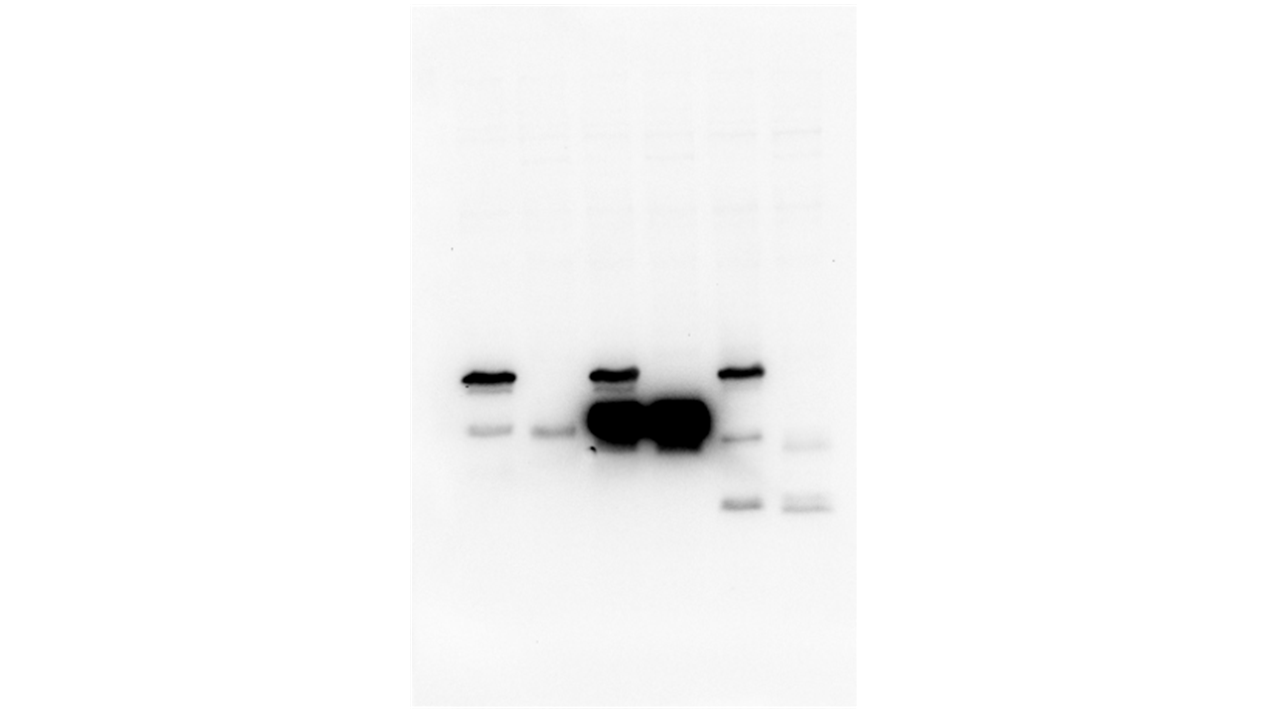

Supplement: Figure 6—figure supplement 1—source data 1. [file elife-106975-fig6-figsupp1-data1.zip › Figure 6-figure supplement 1D, F_FlagChannel.tif]

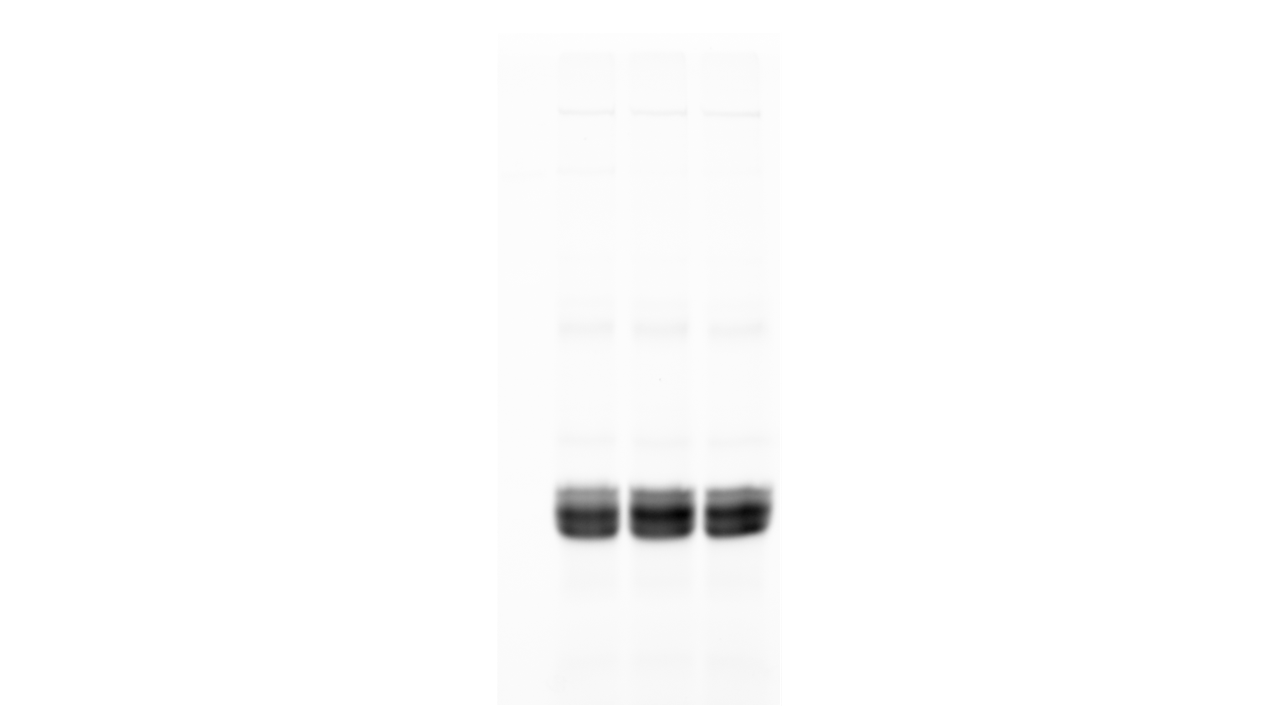

Supplement: Figure 6—figure supplement 2—source data 1. [file elife-106975-fig6-figsupp2-data1.zip › Figure 6-figure supplement 2B_Fluor.tif]

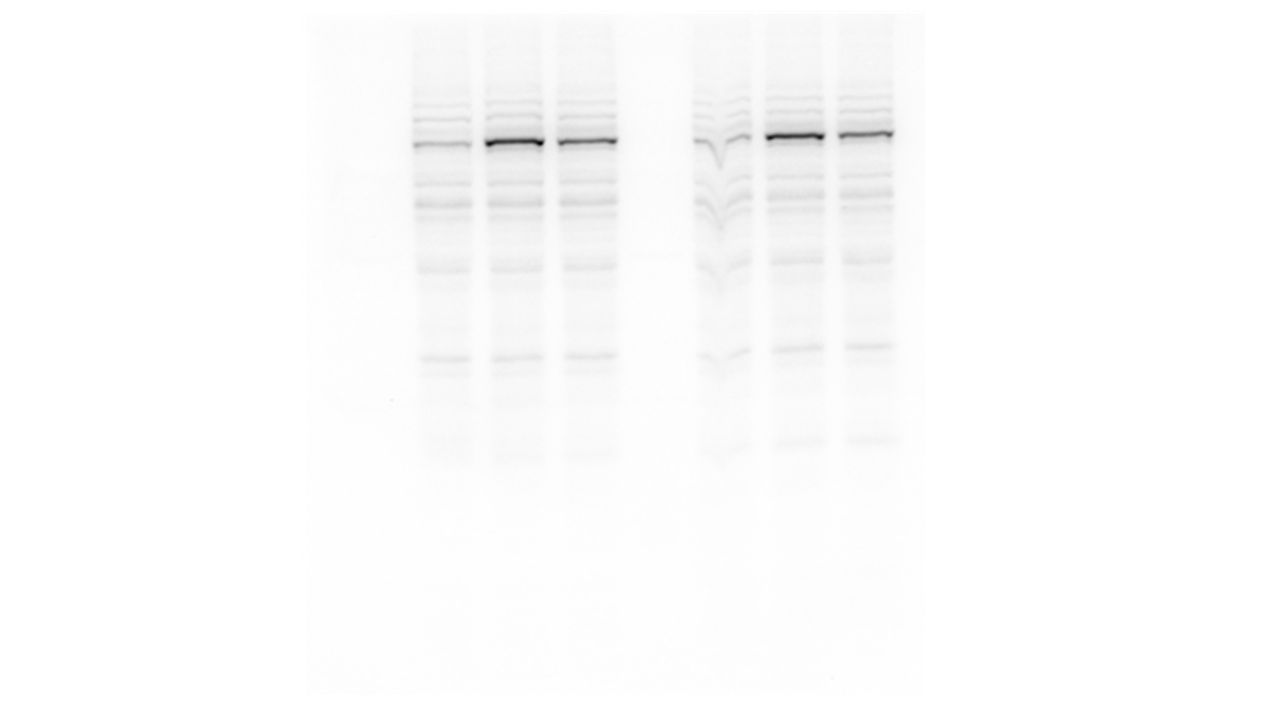

Supplement: Figure 6—figure supplement 2—source data 1. [file elife-106975-fig6-figsupp2-data1.zip › Figure 6-figure supplement 2A, B_GFPChannel.tif]

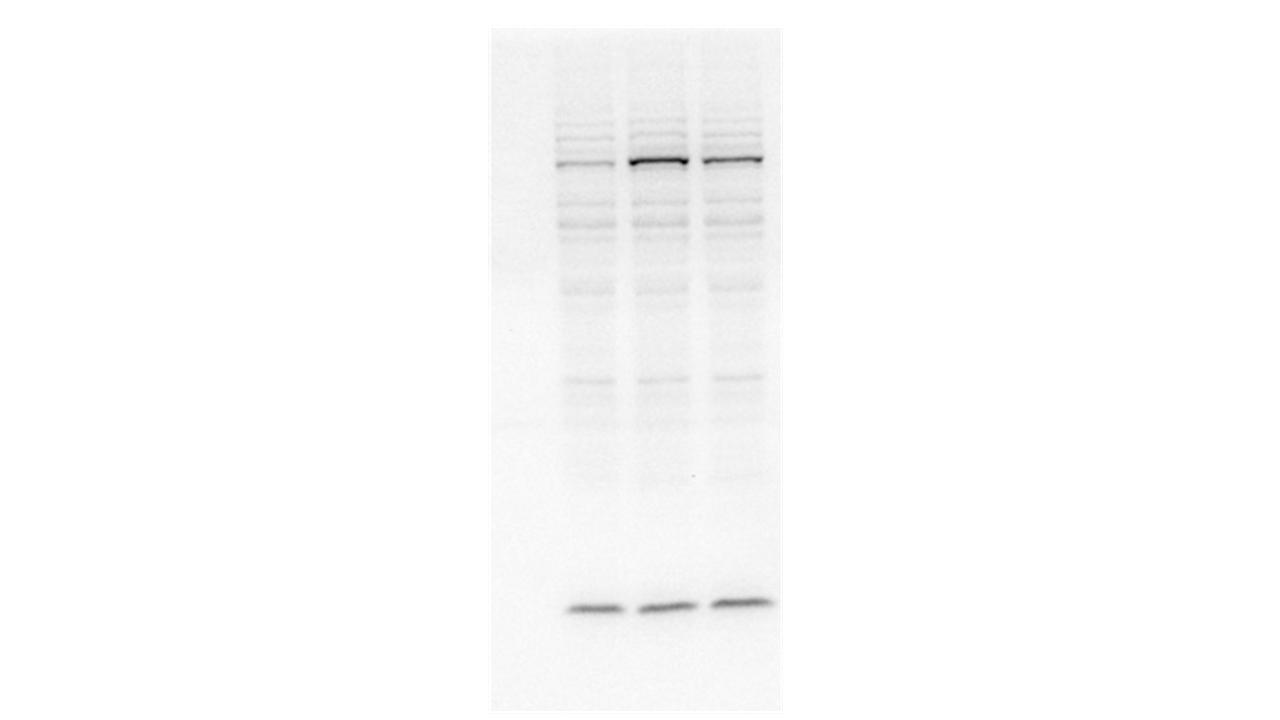

Supplement: Figure 6—figure supplement 2—source data 1. [file elife-106975-fig6-figsupp2-data1.zip › Figure 6-figure supplement 2A_FlagChannel.tif]

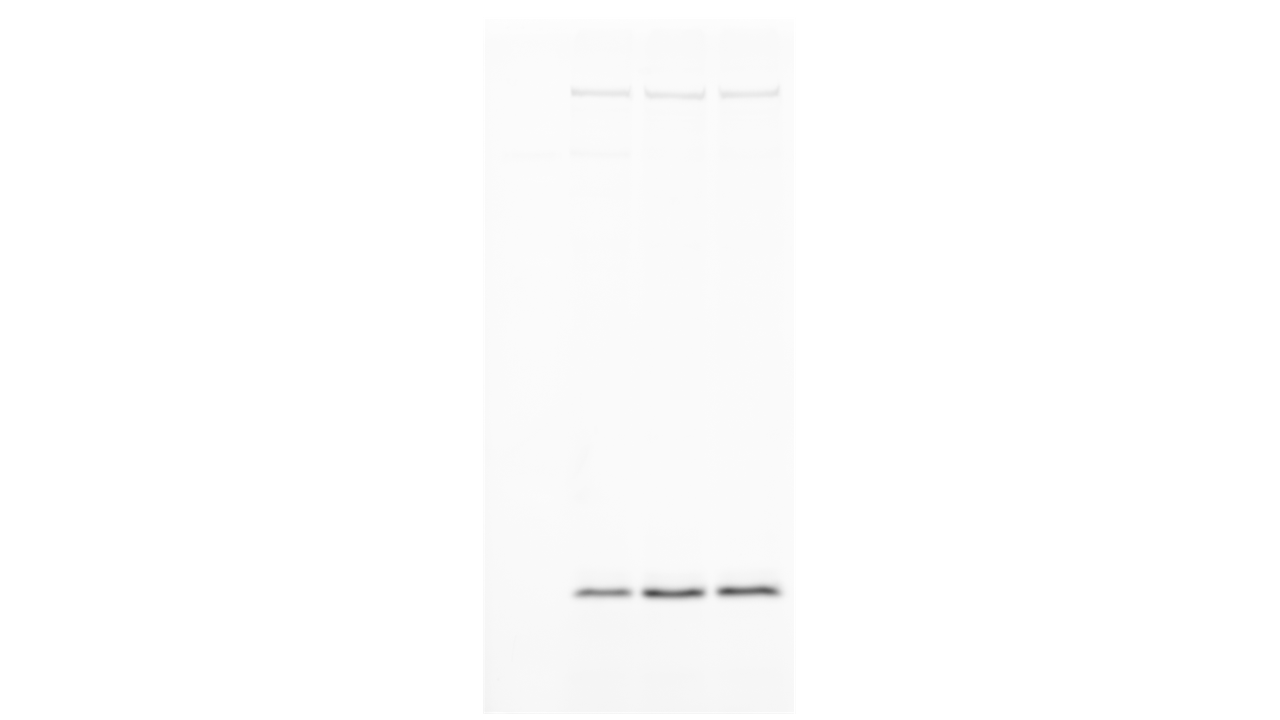

Supplement: Figure 6—figure supplement 2—source data 1. [file elife-106975-fig6-figsupp2-data1.zip › Figure 6-figure supplement 2A_Fluor.tif]

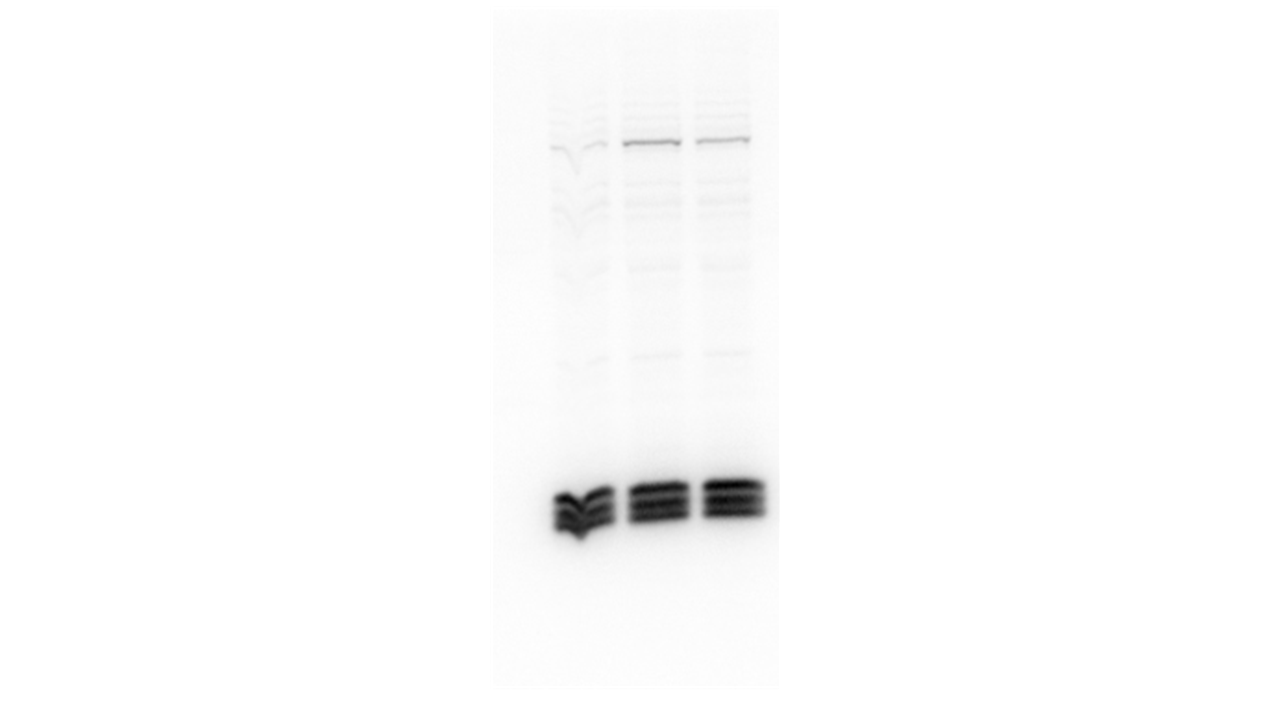

Supplement: Figure 6—figure supplement 2—source data 1. [file elife-106975-fig6-figsupp2-data1.zip › Figure 6-figure supplement 2B_FlagChannel.tif]
