# Supplementary material for: Cryo-EM structure revealed a novel F-actin binding motif in a Legionella pneumophila lysine fatty acyltransferase
Source: eLife. 2026 Jan 28;14:RP106975. doi: 10.7554/eLife.106975 (PMC12851578; doi:10.7554/eLife.106975)
Supplement: Figure 1—source data 2. [file elife-106975-fig1-data2.zip › Figure1_sourcedata 2.pdf]

1D

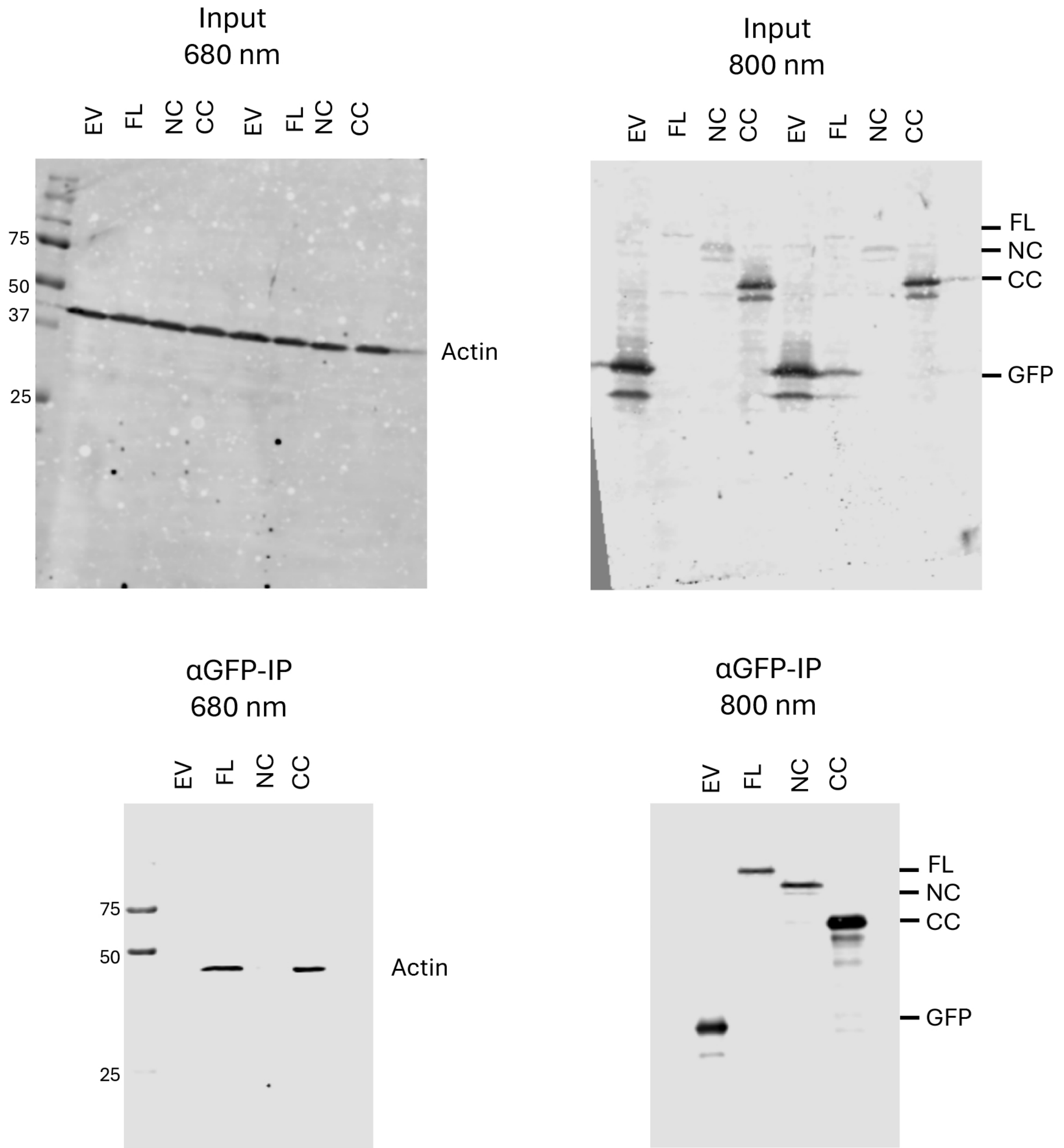

1E

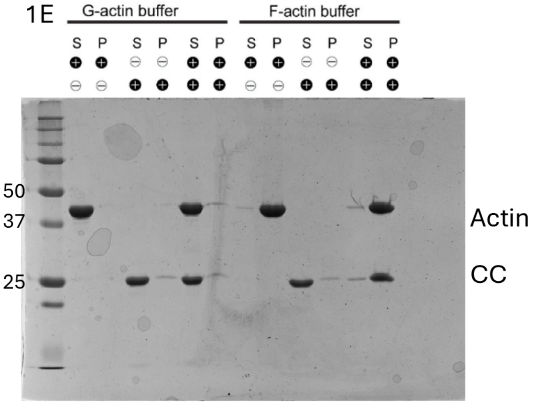

1F

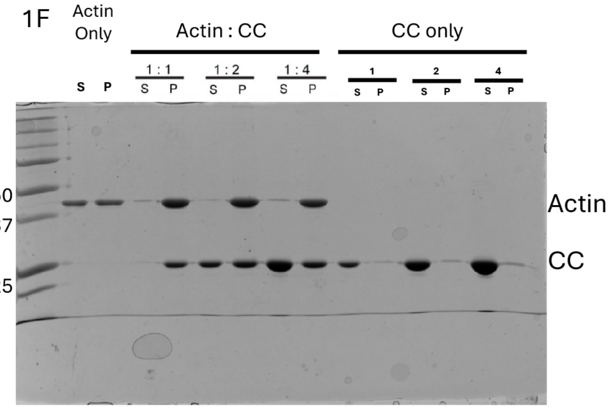

Figure 1, Source Data 1. Original Western Blots and SDS-PAGE corresponding to Figure 1D, E and F.
