# Supplementary material for: Cryo-EM structure revealed a novel F-actin binding motif in a Legionella pneumophila lysine fatty acyltransferase
Source: eLife. 2026 Jan 28;14:RP106975. doi: 10.7554/eLife.106975 (PMC12851578; doi:10.7554/eLife.106975)
Supplement: Figure 3—source data 2. [file elife-106975-fig3-data2.zip › Figure 3 source data 2.pdf]

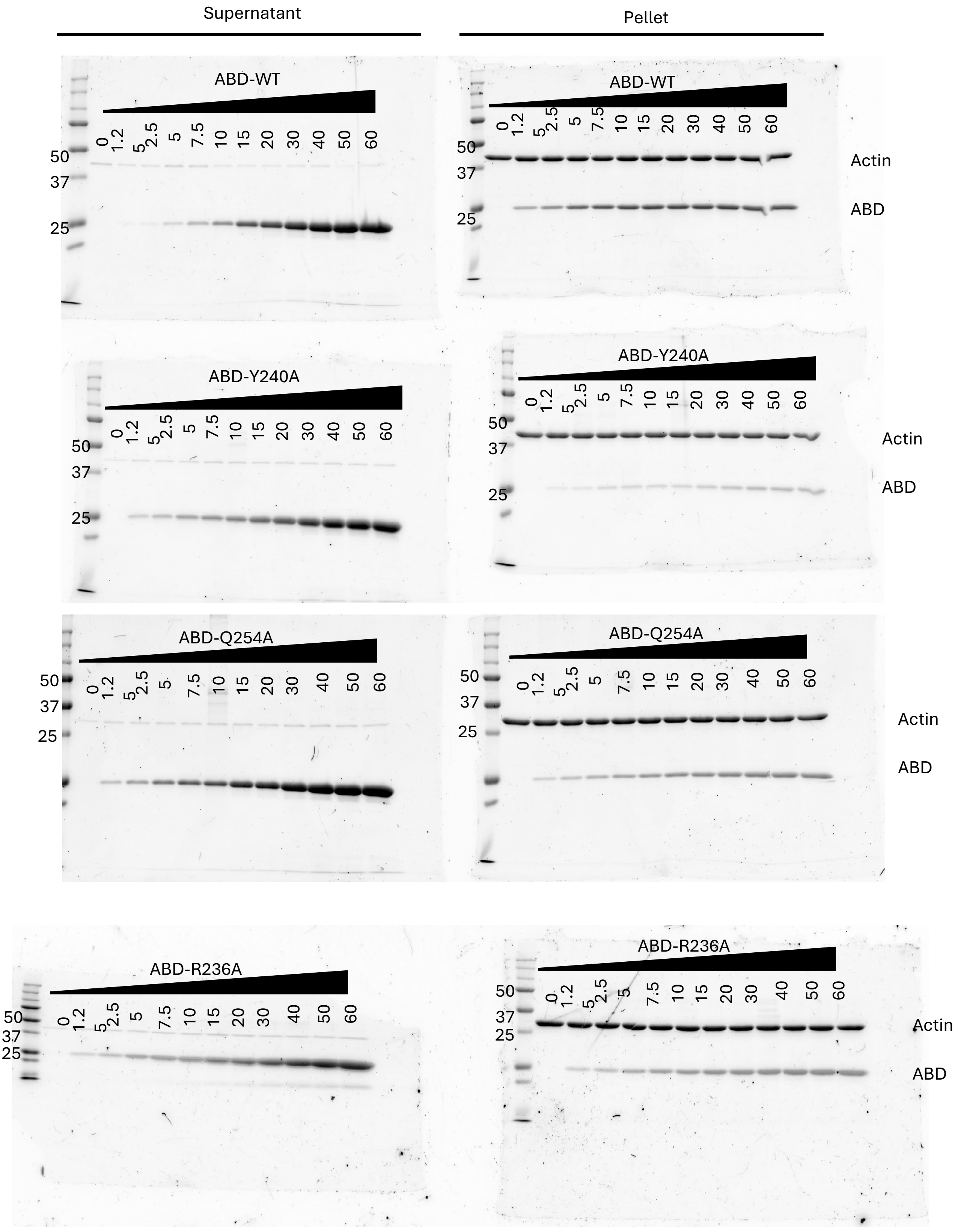

Figure 3, source data 1. SDS-PAGE of co-sedimentation assay. Replicate one of three.

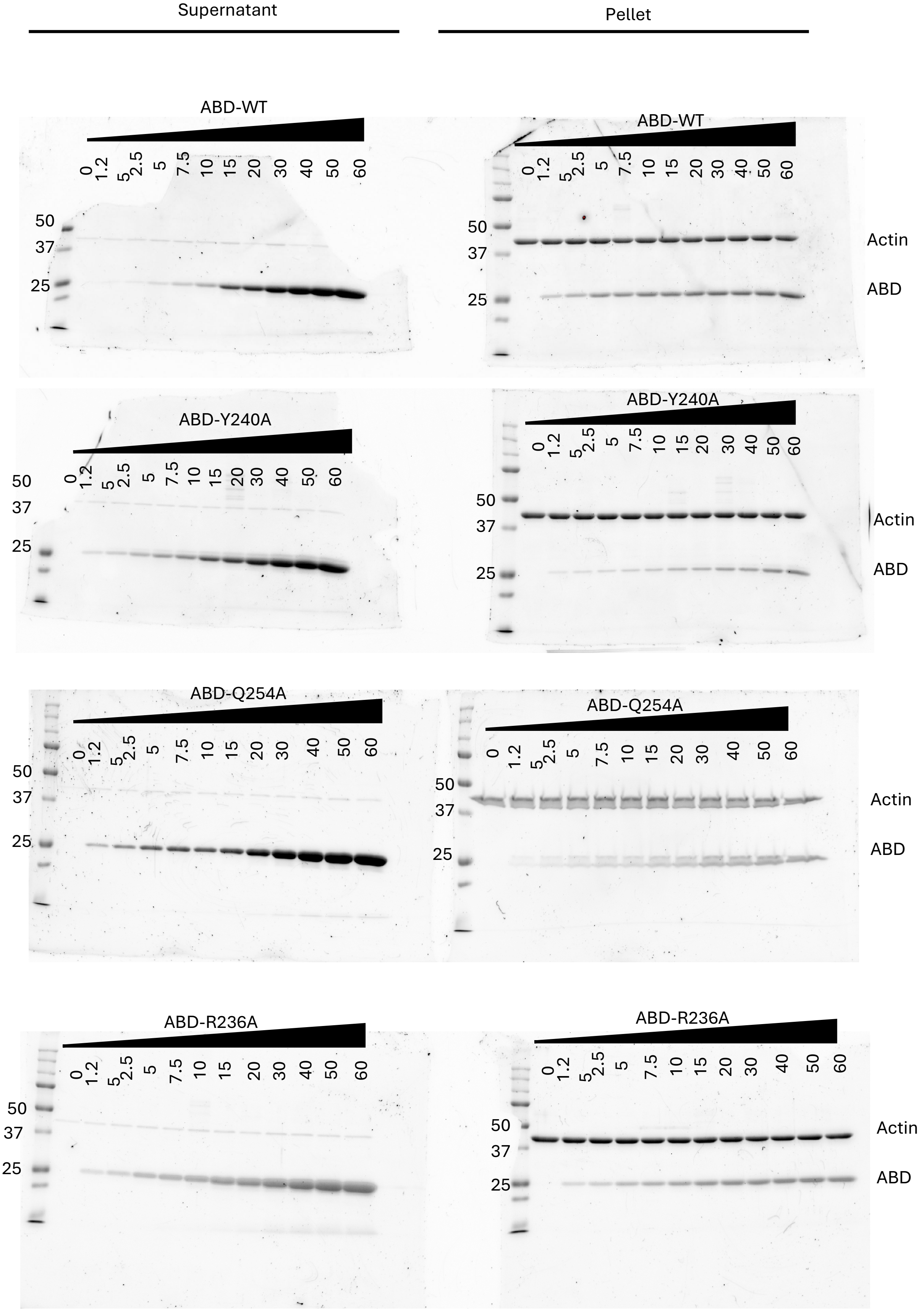

Figure 3, source data 1. SDS-PAGE of co-sedimentation assay. Replicate two of three.

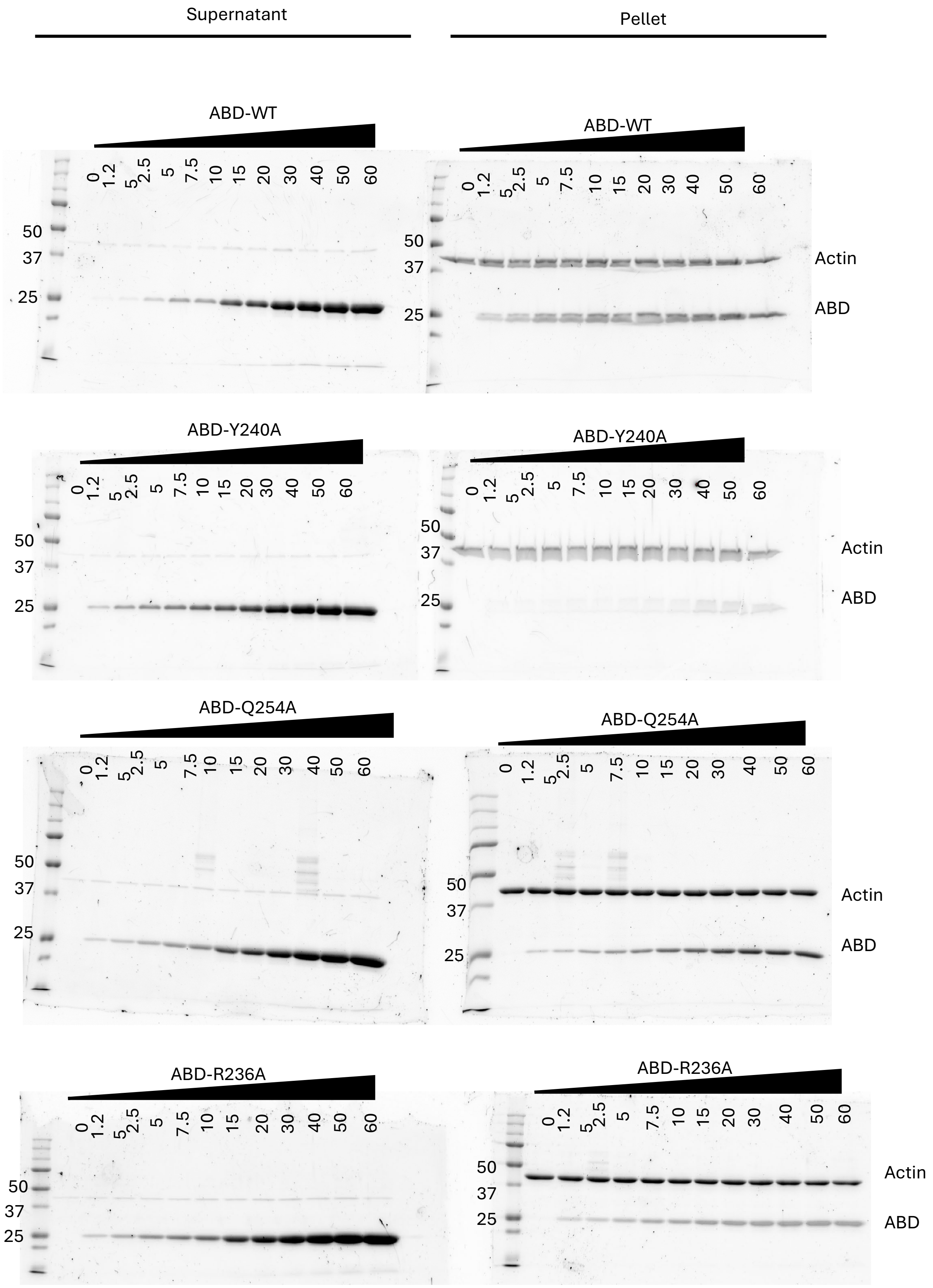

Figure 3, source data 1. SDS-PAGE of co-sedimentation assay. Replicate three of three.
