# Supplementary material for: Cryo-EM structure revealed a novel F-actin binding motif in a Legionella pneumophila lysine fatty acyltransferase
Source: eLife. 2026 Jan 28;14:RP106975. doi: 10.7554/eLife.106975 (PMC12851578; doi:10.7554/eLife.106975)
Supplement: Figure 6—source data 2. [file elife-106975-fig6-data2.zip › Figure 6 Source data 2.pdf]

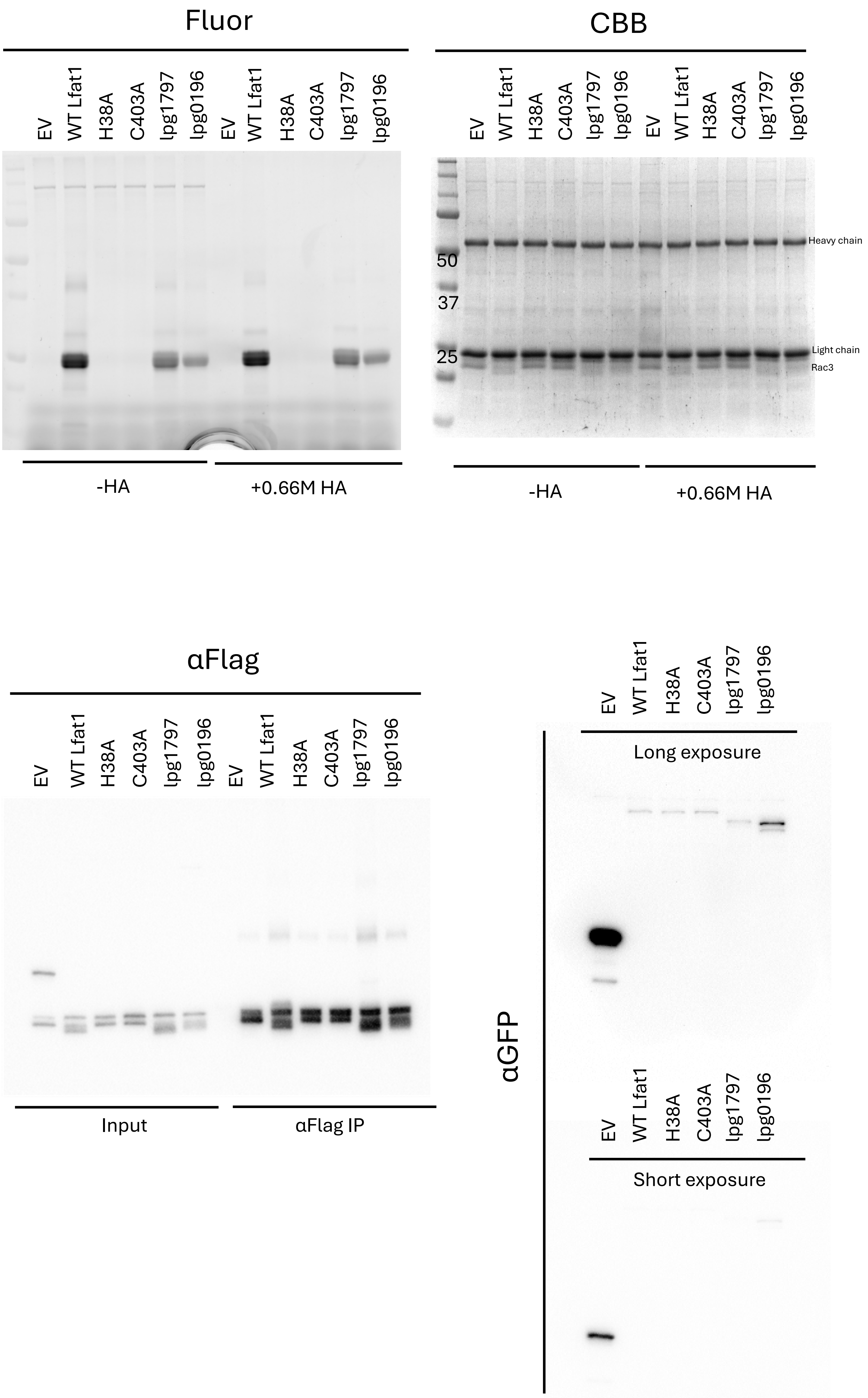

Figure 6 Source Data 1. Original membranes corresponding to Figure 6B. EV = GFP empty vector, lpg1797 and lpg0196 are two homologs of Lfat1 that were not within the scope of this study.
