# Supplementary material for: Cryo-EM structure revealed a novel F-actin binding motif in a Legionella pneumophila lysine fatty acyltransferase
Source: eLife. 2026 Jan 28;14:RP106975. doi: 10.7554/eLife.106975 (PMC12851578; doi:10.7554/eLife.106975)
Supplement: Figure 6—figure supplement 1—source data 2. [file elife-106975-fig6-figsupp1-data2.zip › Figure 6-figure supplemtal 1 data source 2.pdf]

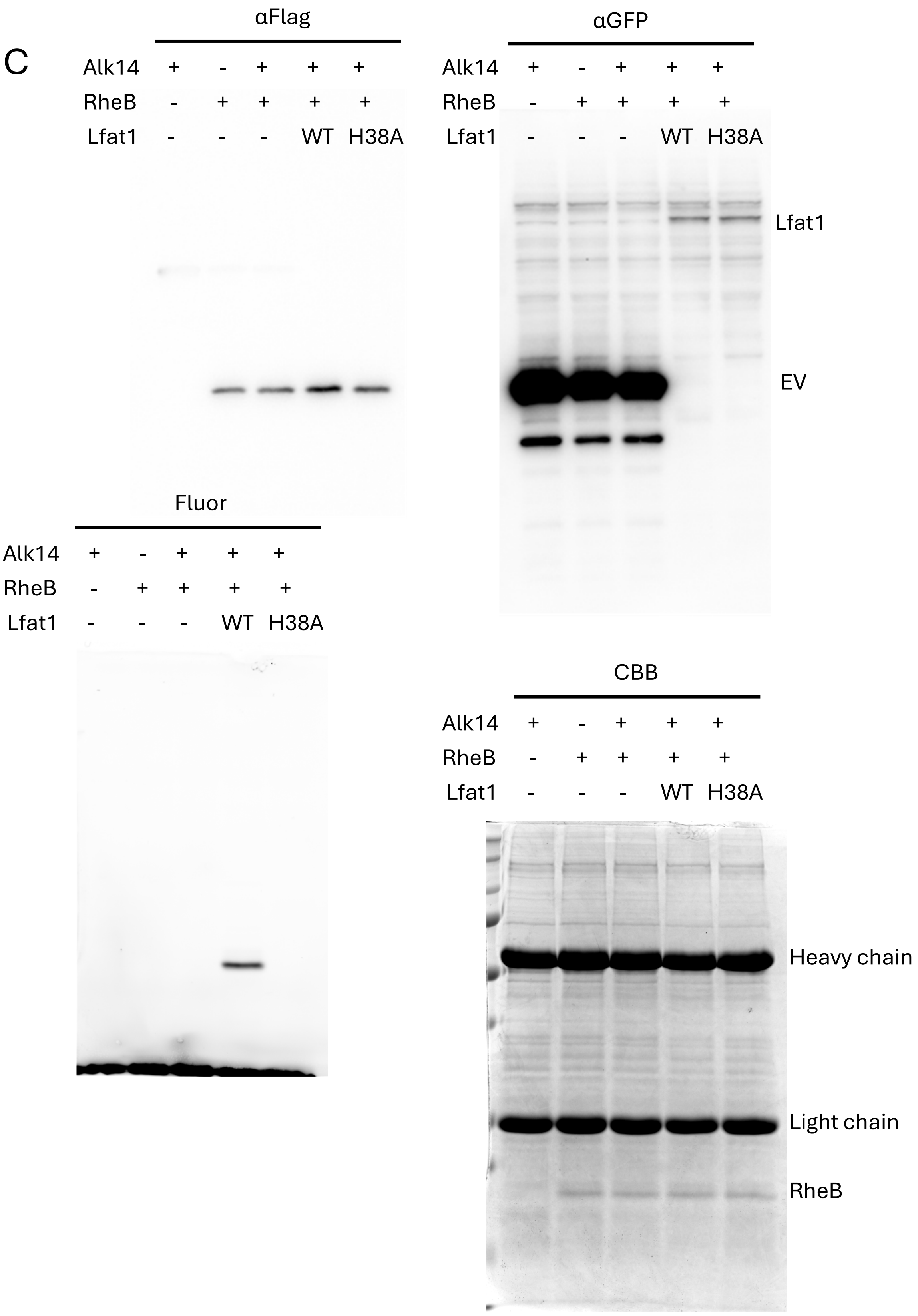

Figure 6-figure supplement 1, Source data 2. Original membranes corresponding to 6-figure supplement 1C (Flag-RheB).

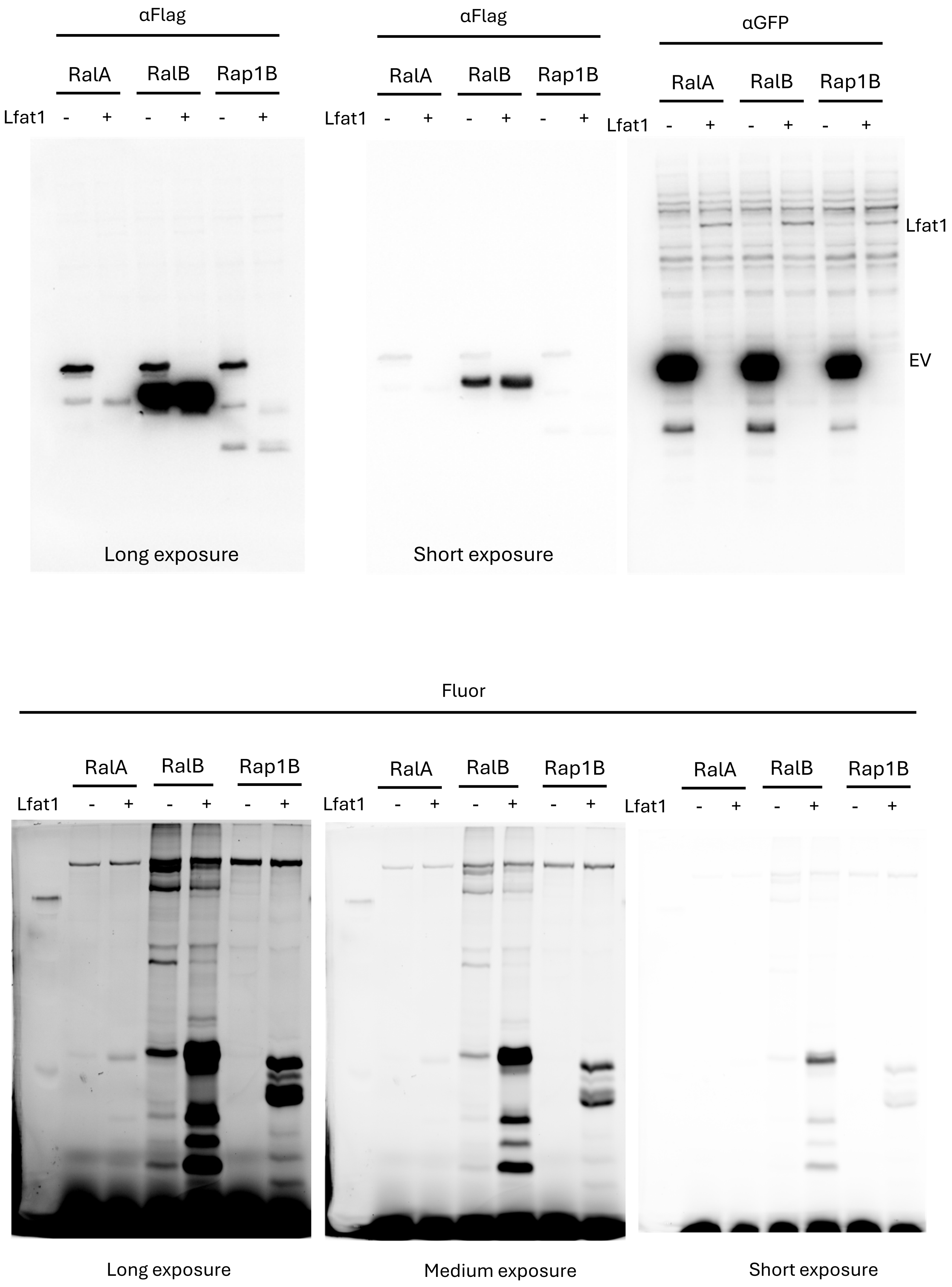

Figure 6-figure supplement 1, Source data 2. Original membranes corresponding to 6-figure supplement 1D (Flag-RalA), E (Flag-RalB), and F (Flag-Rap1B).
