# Supplementary material for: Cryo-EM structure revealed a novel F-actin binding motif in a Legionella pneumophila lysine fatty acyltransferase
Source: eLife. 2026 Jan 28;14:RP106975. doi: 10.7554/eLife.106975 (PMC12851578; doi:10.7554/eLife.106975)
Supplement: Figure 6—figure supplement 2—source data 2. [file elife-106975-fig6-figsupp2-data2.zip › Figure 6-figure supplement 2 source data 2.pdf]

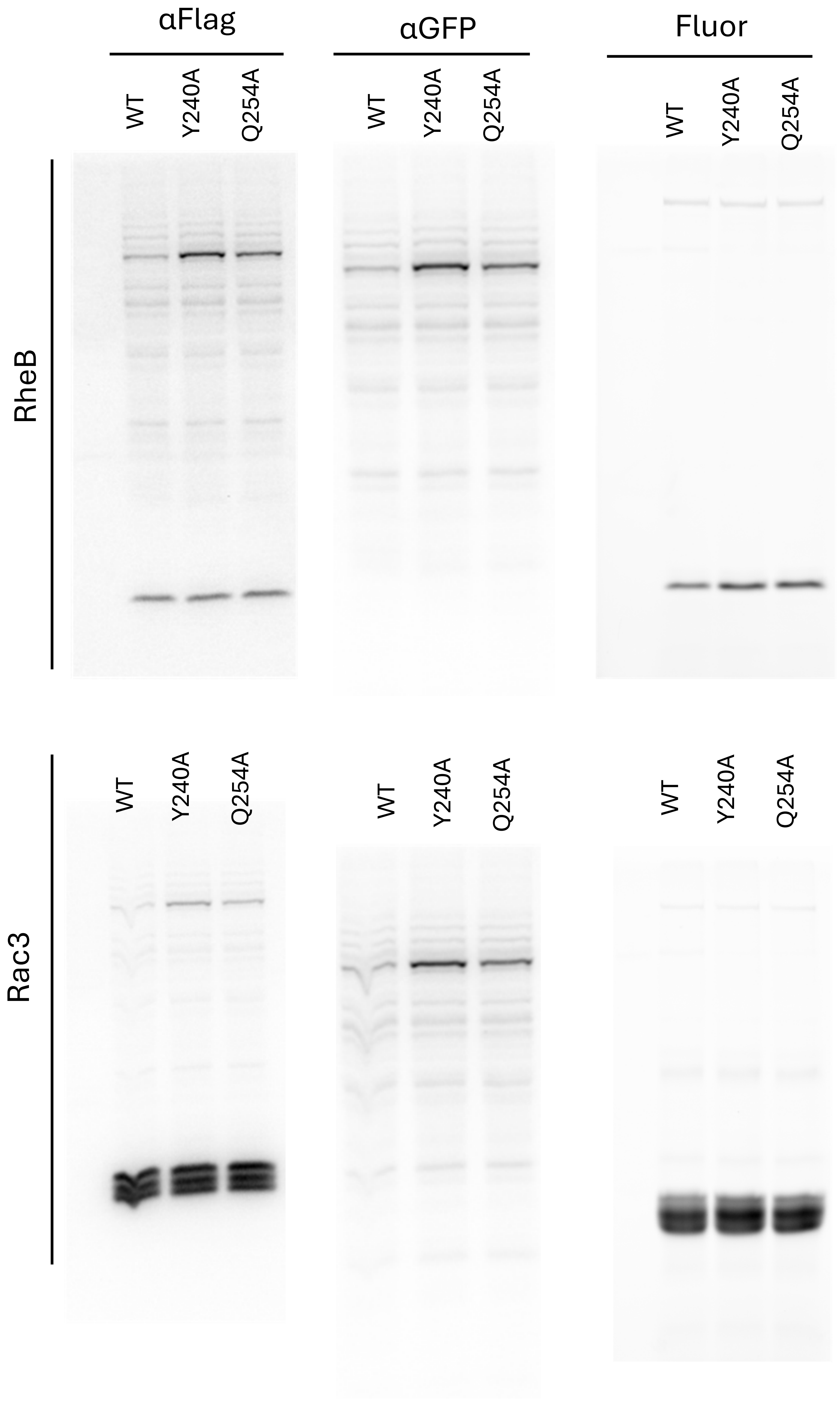

Figure 6-figure supplement 2 source Data 1. Original membranes for Supplemental Figure A (Flag-RheB) and B (Flag-Rac3)
